# Angiotensin II type-1 receptor (AT_1_R) regulates expansion, differentiation, and functional capacity of antigen-specific CD8^+^ T cells

**DOI:** 10.1038/srep35997

**Published:** 2016-10-26

**Authors:** João Luiz Silva-Filho, Celso Caruso-Neves, Ana Acacia Sá Pinheiro

**Affiliations:** 1 Instituto de Biofísica Carlos Chagas Filho, Universidade Federal do Rio de Janeiro, Rio de Janeiro, 21941–902, Brazil; 2 Instituto Nacional de Ciência e Tecnologia em Biologia e Bioimagem, Conselho Nacional de Desenvolvimento Científico e Tecnológico/MCT, Rio de Janeiro, 21941–902, Brazil; 3 Instituto Nacional para Pesquisa Translacional em Saúde e Ambiente na Região Amazônica, Conselho Nacional de Desenvolvimento Científico e Tecnológico/MCT, Rio de Janeiro, 21941–902, Brazil

## Abstract

Angiotensin II (Ang II) and its receptor AT_1_ (AT_1_R), an important effector axis of renin-angiotensin system (RAS), have been demonstrated to regulate T-cell responses. However, these studies characterized Ang II and AT_1_R effects using pharmacological tools, which do not target only Ang II/AT_1_R axis. The specific role of AT_1_R expressed by antigen-specific CD8^+^ T cells is unknown. Then we immunized transgenic mice expressing a T-cell receptor specific for SIINFEKL epitope (OT-I mice) with sporozoites of the rodent malaria parasite *Plasmodium berghei* expressing the cytotoxic epitope SIINFEKL. Early priming events after immunization were not affected but the expansion and contraction of AT_1_R-deficient (AT_1_R^−/−^) OT-I cells was decreased. Moreover, they seemed more activated, express higher levels of CTLA-4, PD-1, LAG-3, and have decreased functional capacity during the effector phase. Memory AT_1_R^−/−^ OT-I cells exhibited higher IL-7Rα expression, activation, and exhaustion phenotypes but less cytotoxic capacity. Importantly, AT_1_R^−/−^ OT-I cells show better control of blood parasitemia burden and ameliorate mice survival during lethal disease induced by blood-stage malaria. Our study reveals that AT_1_R in antigen-specific CD8^+^ T cells regulates expansion, differentiation, and function during effector and memory phases of the response against *Plasmodium*, which could apply to different infectious agents.

In the last decade, new components, local tissue production, and functions of the renin-angiotensin system (RAS) have been unraveled^1^,^2^,^3^,^4^. Our group and others have focused on describing the role of RAS effector molecules, especially angiotensin II (Ang II) and its receptor AT_1_ (AT_1_R), in the regulation of adaptive immune response^5^,^6^,^7^,^8^,^9^,^10^,^11^,^12^,^13^,^14^,^15^,^16^,^17^,^18^,^19^,^20^,^21^. Ang II has been proposed to have pro-inflammatory effects, via AT_1_R^8^,^9^,^10^,^11^,^12^,^13^,^15^,^16^,^17^,^18^,^19^,^20^, however the cellular mechanisms underlying the role of AT_1_R in the regulation of antigen-specific T cells in different pathologies are not clear yet.

It has been demonstrated that T cells possess a functional RAS and are capable of producing Ang II^10^,^11^,^12^,^13^,^14^,^20^,^21^. In these cells, Ang II promotes proliferation, differentiation, effector function, adhesion, migration, and acts as a co-stimulatory molecule important to T-cell activation, and all these effects are mediated by AT_1_R binding^5^,^6^,^7^,^9^,^10^,^11^,^12^,^13^,^14^,^15^,^20^,^21^. Importantly, AT_1_R expression is upregulated in T cells during activation *in vitro* and *in vivo*, for instance during the blood stage of *Plasmodium berghei* ANKA (PbA) infection, strengthening the importance of this receptor for T-cell response^11^,^12^,^13^,^15^. In this regard, AT_1_R is involved in the higher production of pro-inflammatory cytokines by CD4^+^ T cells and perforin by CD8^+^ T cells, and increased capacity to adhere and migrate through upregulation of adhesion molecules and chemokine receptors^12^,^13^. AT_1_R is also involved in cerebral edema and the behavioral impairment observed during PbA infection, and these could be a result of Ang II-induced CD8^+^ T-cell sequestration in the brain via AT_1_R^13^. Thus, based on the critical role that CD8^+^ T cells play in protective or harmful responses in different conditions, it is important to understand how the Ang II/AT_1_R axis regulates the response of these cells. However, most of the previous studies used pharmacological tools, and the observed effects may not always be due to a specific receptor blockade. In addition, there is no clear evidence regarding the role of AT_1_R expressed by antigen-specific CD8^+^ T cells regulating their response against pathogens during effector or even memory phases, which requires further exploration.

In the context of malaria, CD8^+^ T cells play a critical protective role during the liver stage^22^,^23^. These cells become activated soon after exposure to parasites and their response quickly increases following a narrow regulated program^24^,^25^,^26^. The effector response is detectable 24 h after immunization^25^, followed by accelerated expansion of antigen-specific CD8^+^ T cells, reaching a peak around 5 days after priming^25^. On days 6–8 after immunization, a sudden contraction occurs, probably due to programmed cell death of up to 80% of activated cells, restoring homeostasis^25^,^26^. After this fast contraction phase, the antigen-specific CD8^+^ T-cell population stabilizes and starts the formation of memory cells around day 15 after priming^24^. The development and survival of this population depends on different cytokines secreted by CD4^+^ T cells, such as IL-2, IL-4, IL-7 and IL-15, which inhibit apoptosis^24^,^27^,^28^,^29^,^30^. In addition, these cytokines promote differentiation of sub-populations of memory cells, which acquire a definitive phenotype around 20 days after immunization^24^. Given the large number of other molecules produced by antigen-presenting cells (APCs) and CD4^+^ T cells, such as Ang II, and receptors upregulated in CD8^+^ T cells during this response, such as AT_1_R, the Ang II/AT_1_R axis could also be important in the expansion, differentiation, and functional capacity of effector and memory CD8^+^ T cells.

In the current study, we evaluated the role of AT_1_R expressed in antigen-specific CD8^+^ T cells in their expansion, differentiation, and function during the response induced by immunization of mice with attenuated sporozoites of *Plasmodium berghei*. We used wild-type (WT; AT_1_R^+/+^) and AT_1_R^−/−^ CD8^+^ T cells from ovalbumin (OVA)-specific TCR transgenic mice (OT-I), which allows the response of SIINFEKL-specific CD8^+^ T-cell population to be monitored^31^,^32^,^33^,^34^,^35^,^36^,^37^. For immunization, we used radiation-attenuated sporozoites (γ-spz) of *Plasmodium berghei CS*^*5M*^, which express the OVA peptide, SIINFEKL, replacing the natural epitope from the circumsporozoite (CS) protein^34^,^35^,^36^,^37^. This system provides a clean experimental set up to evaluate the role of AT_1_R in antigen-specific CD8^+^ T cells during the effector and memory phases of the response.

Our results revealed that clonal expansion and contraction of AT_1_R^−/−^ OT-I cells were lower in magnitude compared with AT_1_R^+/+^ OT-I cells. AT_1_R^−/−^ cells showed stronger activation and exhaustion profiles along with lower polyfunctional capacity compared with AT_1_R^+/+^ cells during the effector phase. During the memory development phase, antigen-specific AT_1_R^−/−^ OT-I cells still showed higher expression of activation, central memory markers but increased polyfunctional capacity, in contrast to AT_1_R^+/+^ OT-I control. In addition, lack of AT_1_R expression promotes a larger memory population with lower cytotoxic activity. Genetic ablation of AT_1_R in parasite-specific CD8^+^ T cells also improves control of the parasite burden and survival of mice during lethal disease induced by blood-stage PbA infection. Our study reveals different roles of AT_1_R in antigen-specific CD8^+^ T cells during the effector and memory phases of the response against *Plasmodium*.

## Results

### AT_1_R is important to the expansion of antigen-specific CD8^+^ T cells

**Figure 1:**
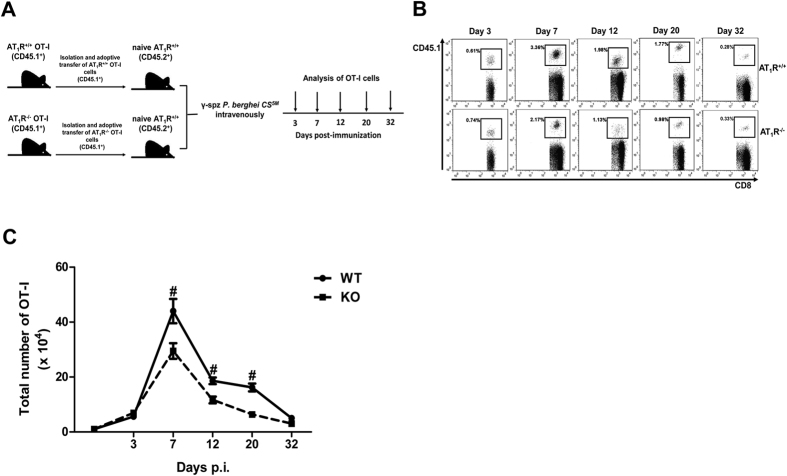
AT_1_R is important to the expansion of antigen-specific CD8^+^ T cells. AT_1_R^+/+^ or AT_1_R^−/−^ OT-I cells (CD8^+^ CD45.1^+^) recovered from the spleen of immunized recipient mice (CD45.2^+^) were analyzed on days 0, 3, 7, 12, 20, and 32 post immunization. (**A**) Schematics of the experimental design. 1 × 10^4^ Naive AT_1_R^+/+^ or AT_1_R^−/−^ OT-I cells (CD8^+^CD45.1^+^) were adoptively transferred to WT C57BL/6 mice (CD45.2^+^) recipients 1 day before intravenous inoculation with 1 × 10^λ^ γ-irradiated *P. berghei CS*^*5M*^ sporozoites. Mice were euthanized at the indicated time points for recovery and analysis of OT-I cells. (**B**) Representative CD8^+^CD45.1^+^(OT-I cells) plots gated on total lymphocytes. Percentages represent the proportion of OT-I cells (CD8^+^CD45.1^+^) among the total CD8^+^ T cells per spleen, recovered at days 3, 7, 12, 20, and 32 p.i. The gating strategy used for flow cytometry analysis is indicated in the Materials and Methods section. (**C**) Total number of AT_1_R^+/+^ (filled circle; continued line) or AT_1_R^−/−^ (filled square; broken line) OT-I cells per spleen at days 3 (p = 0.136), 7 (*p = 0.044), 12 (*p = 0.003), 20 (*p = 0.0002), and 32 (p = 0.129) post inoculation, calculated as the frequencies obtained by CD8^+^ CD45.1^+^ staining, multiplied by the total number of cells obtained after spleen excision. Data are means ± SEM of 4 mice per group and are representative of 3 independent experiments with similar results for each indicated time point. Abbreviations: AT_1_R, angiotensin II type 1 receptor; WT, wild-type.

To determine the importance of AT_1_R in regulating the antigen-specific CD8^+^ T-cell response, we first evaluated the role of AT_1_R in the primary clonal expansion and homeostatic contraction of antigen-specific CD8^+^ T cells induced by immunization with *P. berghei* CS^5M^ γ-spz. Naive AT_1_R^+/+^ or AT_1_R^−/−^ OT-I cells (CD45.1^+^) were adoptively transferred into H-2k^b^ C57BL/6 mice (CD45.2^+^) and 24 h later the recipient mice were immunized with 10^5^ freshly isolated *P. berghei* CS^5M^ γ-spz, which express the H-2k^b^-restricted peptide SIINFEKL in the CS protein^34^. On days 3, 7, 12, 20, and 32 post immunization (p.i.), OT-I cells were isolated from the spleen, and the percentage and absolute number were determined (Fig. 1A) based on the the gate strategy showed in the Supplementary Fig. S1.

Figure 1 shows a robust expansion of AT_1_R^+/+^ OT-I cells reaching a peak on day 7, and a quick contraction was observed thereafter. After day 12, the magnitude of the response is stabilized and remains unchanged up to day 20, when decreases occur until day 32 (Fig. 1B,C). AT_1_R^−/−^ OT-I cells also expand until day 7 followed by contraction. However, the number of AT_1_R^−/−^ OT-I cells at the peak of expansion and at day 12 p.i. is lower than the number of AT_1_R^+/+^ OT-I cells, indicating the importance of AT_1_R in the expansion of antigen-specific CD8^+^ T cells during the effector phase (Fig. 1B,C). Moreover, the number of AT_1_R^−/−^ OT-I cells is still lower at day 20 p.i. (Fig. 1B,C). In parallel, analysis in non-immunized mice (naive) revealed that, without immunization, the non-activated OT-1 cells disappear over time (Supplementary Fig. S2), confirming that stimulation is dependent on the presence of the antigen.

**Figure 2:**
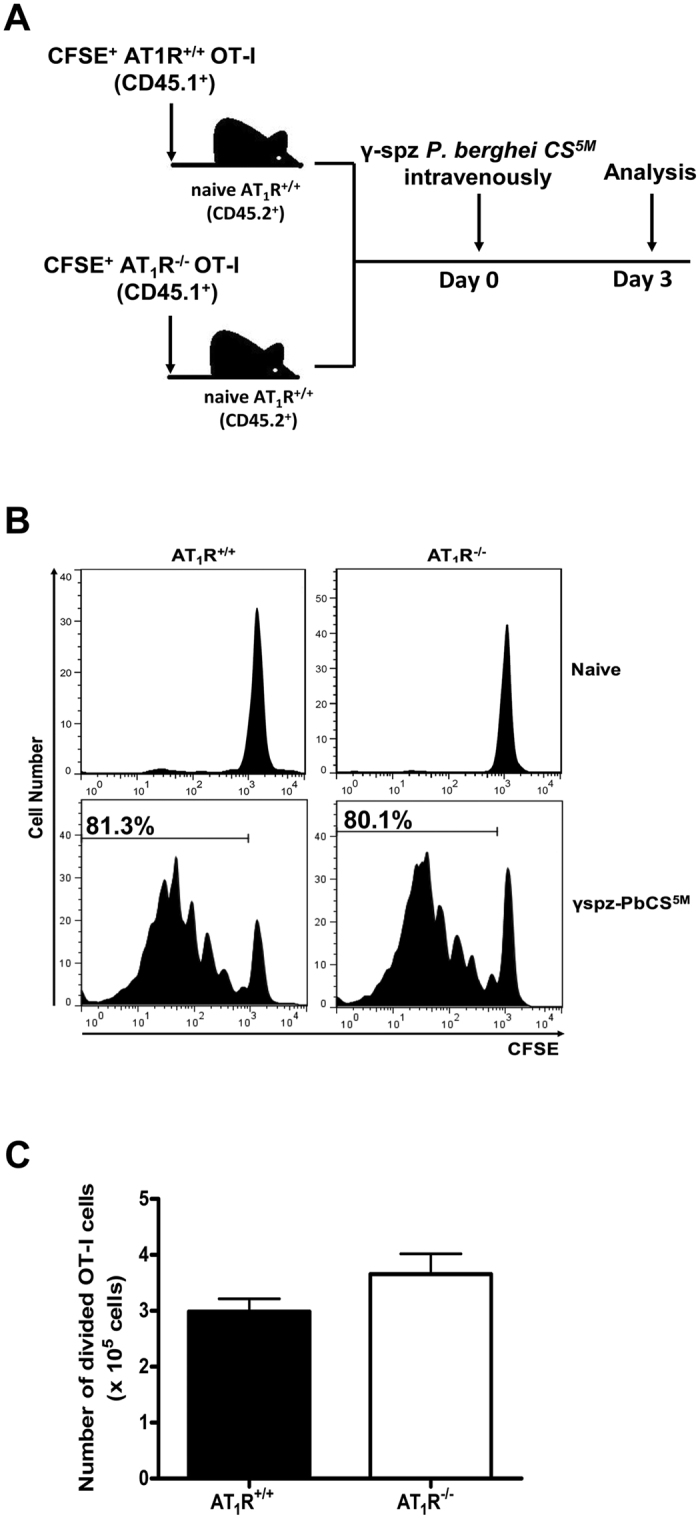
AT_1_R does not affect early proliferation (up to 72 h) of antigen-specific CD8^+^ T cells. (**A**) Schematics of the experimental design. WT **C57BL/6** mice (CD45.2^+^) received 2 × 10^6^ CFSE-labeled naive AT_1_R^+/+^ and AT_1_R^−/−^ OT-I cells (CD8^+^CD45.1^+^) 1 day before inoculation with irradiated *P. berghei* CS^5M^ sporozoites. (**B**) Mice were euthanized 3 days later, and CFSE dilutions on CD8^+^CD45.1^+^ T cells (OT-I cells) harvested from the spleen of non-imunized (naïve) and γ–spz immunized mice were analyzed by flow cytometry. Histograms represent OT-I cells taken from the spleen. (**C**) Number of divided OT-I cells 3 days after γ-spz inoculation, calculated by the frequency of CFSE^lo^CD8^+^CD45.1^+^ cells in the spleen multiplied by the total number of cells obtained after spleen excision (p = 0.139). Data are means ± SEM of 4 mice per group and are representative of 3 independent experiments with similar results for each indicated time point. Abbreviation: CFSE, carboxyfluorescein diacetate succinimidyl ester.

It is known that clonal expansion initiates after cell differentiation, and an increase in the total number of antigen-specific cells is detected only at 48–72 h after spz immunization^25^. Thus, to understand the lower expansion of AT_1_R^−/−^ OT-I cells, we tested if AT_1_R plays a key role in triggering signals to initiate clonal expansion by detecting cellular proliferation at 72 h after immunization. To do so, carboxyfluorescein succinimidyl ester (CFSE)-stained naive AT_1_R^+/+^ and AT_1_R^−/−^ OT-I cells (2 × 10^6^ cells; CD45.1^+^) were adoptively transferred into WT mice (CD45.2^+^). After 24 h, recipient mice were inoculated with irradiated *P. berghei* CS^5M^ sporozoites and 72 h after immunization, OT-I cells were isolated from the spleen and quantified by CFSE dilution using flow cytometry (Fig. 2). Early proliferation (up to 72 h) was not affected by AT_1_R deficiency because the rate of CFSE dilution and the number of divided OT-I were the same for AT_1_R^+/+^ and AT_1_R^−/−^ OT-I cells (Fig. 2) in γ-spz-immunized mice. In non-immunized mice (naive), OT-I cells do not proliferate.

### AT_1_R upregulates the IL-2/IL2-R axis in antigen-specific CD8^+^ T cells during the effector phase

A possible mechanism to explain the lower expansion of AT_1_R^−/−^ OT-I cells from 3 days after immunization is the alteration in the expression of the IL-2R subunits and/or IL-2 production, because IL-2 signaling is important for CD8^+^ T-cell proliferation as shown in a number of models including γ-spz immunization^24^,^29^,^30^.

**Figure 3:**
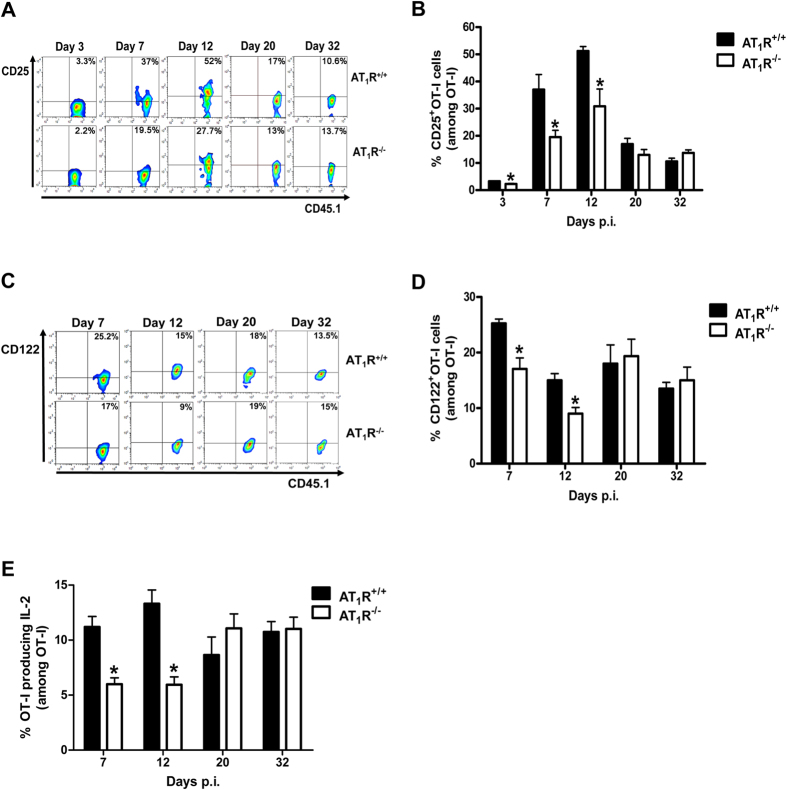
AT_1_R upregulates IL-2 production and IL-2R expression in antigen-specific CD8^+^ T cells during the effector phase. 1 × 10^4^ naive AT_1_R^+/+^ or AT_1_R^−/−^ CD45.1^+^ OT-I cells were adoptively transferred to WT C57BL/6 (CD45.2^+^) recipients 1 day before IV inoculation with 1 × 10^5^ γ-irradiated *P. berghei CS*^*5M*^ sporozoites. IL-2R subunits (CD25 (α-chain) and CD122 (β-chain)) and IL-2 production were evaluated in AT_1_R^+/+^ and AT_1_R^−/−^ OT-I cells (CD8^+^CD45.1^+^) recovered from the spleen of immunized recipient mice (CD45.2^+^) at indicated time points post immunization. The gating strategy used for flow cytometry analysis is indicated in the Materials and Methods section. (**A,B**) Representative dot plots and percentage of CD25^+^ OT-I cells among total OT-I cells in the spleen on days 3 (*p = 0.0289), 7 (*p = 0.0376), 12 (*p = 0.0292), 20 (p = 0.7879), and 32 (p = 0.663) post immunization. (**C,D**) Representative dot plots and percentage of CD122^+^ OT-I cells among total OT-I cells in the spleen on days 7 (*p = 0.0161), 12 (*p = 0.048), 20 (p = 0.2063), and 32 (p = 0.281) post immunization. Data are means ± SEM of 4 mice per group and are pooled from 2 independent experiments with similar results for each indicated day. (**E**) IL-2 production in AT_1_R^+/+^ and AT_1_R^−/−^ and OT-I was evaluated after 4-h *ex vivo* re-stimulation with cognate peptide on days 7 (*p = 0.0007), 12 (*p = 0.007), 20 (p = 0.19), and 32 (p = 0.9887) post immunization. Data are means ± SEM of 4 mice per group and are pooled from 2 independent experiments with similar results for each indicated day. Abbreviations: IL-2, interleukin-2; IL-2R, interleukin-2 receptor.

Figure 3 and Supplementary Figure S3 shows that at day 3, the percentage of CD25^+^ CD8^+^ T cells and expression of CD25, determined by median fluorescence intensity (MFI), is slightly but significantly lower in the AT_1_R^−/−^ OT-I cells. From day 3, there is an increase in the percentage of CD25^+^ AT_1_R^+/+^ OT-I cells and CD25 expression, which reaches a peak at day 12 p.i. (Fig. 3A,B and Supplementary Fig. S3), then CD25 expression decreases during memory development phase, in agreement with the literature and our results using recombinant vaccinina virus expressing OVA (data not shown) (Fig. 3A,B and Supplementary Fig. S3)^30^. In AT_1_R^−/−^ OT-I cells, CD25 expression is also upregulated, but to a lesser extent inasmuch the percentage of CD25^+^ cells and expression are significantly lower in the AT_1_R^−/−^ population up to day 12 p.i. (Fig. 3 and Supplementary Fig. S3). With regard to the percentage of CD122^+^ OT-I cells and CD122 expression, a similar profile to CD25 was observed (Fig. 3C,D and Supplementary Fig. S3). Functional analysis revealed AT_1_R^−/−^ OT-I cells produced considerably lower amounts of IL-2 at days 7 and 12 p.i. in comparison with AT_1_R^+/+^ OT-I cells (Fig. 3E). This is in agreement with the lower expression of IL-2R subunits and lower expansion of AT_1_R^−/−^ OT-I cells from day 3 to day 32. These results indicate the importance of AT_1_R for the expansion of antigen-specific CD8^+^ T cells during the effector phase, which could be correlated to the increased production of IL-2 and upregulation of IL-2R subunits.

### AT_1_R prevents exacerbated activation of antigen-specific CD8^+^ T cells during the effector and memory phases

Next, to verify if AT_1_R plays a role in the activation of antigen-specific CD8^+^ T cells induced by γ-spz immunization, the expression of different T-cell activation surface markers, such as CD69, CD44, CD62L, and CD160, were evaluated at days 7, 12, 20, and 32 p.i.

**Figure 4:**
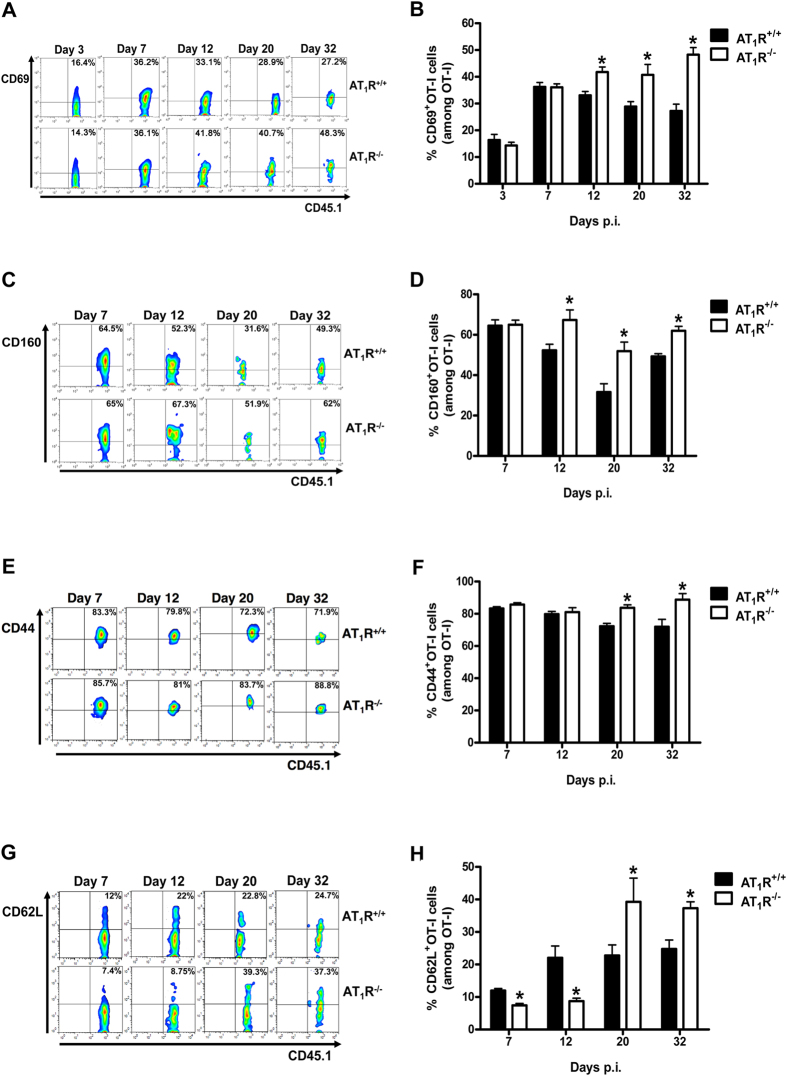
AT_1_R prevents exacerbated activation of antigen-specific CD8^+^ T cells during effector and memory phases. Percentage of cells expressing the markers of activation, CD69, CD160, CD44, and CD62L, were evaluated in AT_1_R^+/+^ and AT_1_R^−/−^ OT-I cells (CD8^+^CD45.1^+^) recovered from the spleen of immunized recipient mice (CD45.2^+^) at indicated time points post immunization. The gating strategy used for flow cytometry analysis is indicated in the Materials and Methods section. (**A,B**) Representative dot plots and percentage of CD69^+^ OT-I cells among total OT-I cells in the spleen on days 3 (p = 0.4255), 7 (p = 0.9451), 12 (*p = 0.0210), 20 (*p = 0.0310), and 32 (*p = 0.0003) post immunization. (**C,D**) Representative dot plots and percentage of CD160^+^ OT-I cells among total OT-I cells in the spleen on days 7 (p = 0.9054), 12 (*p = 0.0287), 20 (*p = 0.0095), and 32 (*p = 0.0069) post immunization. Data are means ± SEM of 4 mice per group and are pooled from 2 independent experiments with similar results for each indicated day. (**E,F**) Representative dot plots and percentage of CD44^+^ OT-I cells among total OT-I cells in the spleen on days 7 (p = 0.2), 12 (p = 0.8425), 20 (*p = 0.0002), and 32 (*p = 0.0296) post immunization. (**G,H**) Representative dot plots and percentage of CD62L^+^ OT-I cells among total OT-I cells in the spleen on days 7 (*p = 0.0009), 12 (*p = 0.0115), 20 (*p = 0.0139), and 32 (*p = 0.0052) post immunization. Data are means ± SEM of 4 mice per group and are pooled of 3 independent experiments with similar results for each indicated day.

As depicted in Figs 4 and S3C, from day 3, expression of CD69 upregulates in both AT_1_R^+/+^ and AT_1_R^−/−^ OT-I cells. However, CD69 frequency and expression become higher in AT_1_R^−/−^ OT-I cells than in AT_1_R^+/+^ OT-I cells from day 12 to day 32 p.i. (Fig. 4A,B and Supplementary Fig. S3C). CD160 expression decreases in AT_1_R^+/+^ OT-I cells from day 7, whereas the frequency of CD160^+^ cells and expression in the AT_1_R^−/−^ OT-I population is higher from day 12 (Fig. 4C,D and Supplementary Fig. S3D).

Similarly, the frequency and expression of CD44 are significantly higher in AT_1_R-deficient cells after 12 days p.i. (Fig. 4E,F and Supplementary Fig. S3E). CD62L, known as L-selectin, characterizes effector cells when its expression is decreased but also represents central memory cells when its expression is increased after T-cell activation^38^,^39^. As observed in the Figs 4 and S3F, the frequency and expression of CD62L is significantly lower in AT_1_R^−/−^ OT-I cells during the effector phase of the response (up to day 12 p.i.). However, during memory development phase (days 20 and 32), CD62L expression and frequency are higher in AT_1_R^−/−^ cells than in AT_1_R^+/+^ cells (Fig. 4G,H and Supplementary Fig. S3F).

The higher expression of CD69, CD44, CD160 and the lower expression of CD62L suggest that AT_1_R^−/−^ OT-I cells are phenotypically more activated than AT_1_R^+/+^ cells during the effector phase (up to day 12). The higher expression of CD44 and CD62L, during memory phase, also indicates that the AT_1_R^−/−^ OT-I cells progress to a central memory phenotype.

In γ-spz immunized mice, OT-I cells are only stimulated by parasites expressing the SIINFEKL epitope^34^,^36^,^37^ but during blood-stage malaria, a low level of non-specific activation has been described^33^. Thus, to check whether non-specific activation could occur, we analyzed the activation status of the endogenous CD8^+^ T cell population (CD8^+^ CD45.1^−^). We reasoned that if non-specific activation of OT-I cells takes place, activation of endogenous CD8^+^ T cells in the presence of γ-irradiated *P. berghei* CS^5M^ sporozoites will also take place. No detectable changes in the absolute number, expression of CD25, CD122, activation, exhaustion, or memory markers were observed in the total endogenous CD8^+^ T cells in comparison with non-immunized recipient mice (Supplementary Figs S2, S4, and S5). These observations confirm that immunization induces stimulation in the transferred CD8^+^ T cells in an antigen-specific manner.

### AT_1_R decreases exhaustion of antigen-specific CD8^+^ T cells

So far, our results indicate that AT_1_R expression affects expansion and activation phenotypes during γ-spz-induced antigen-specific CD8^+^ T-cell response. It is known that on activation, and with progressive differentiation to effector cells, the so-called inhibitory receptors or cellular exhaustion markers are expressed, which function as a negative feedback mechanism and are linked to activation and effector differentiation^40^,^41^. Thus, to evaluate the possible influence of AT_1_R in exhaustion, we characterized the frequency and expression of exhaustion markers, such as PD1, CTLA4, and LAG3.

**Figure 5:**
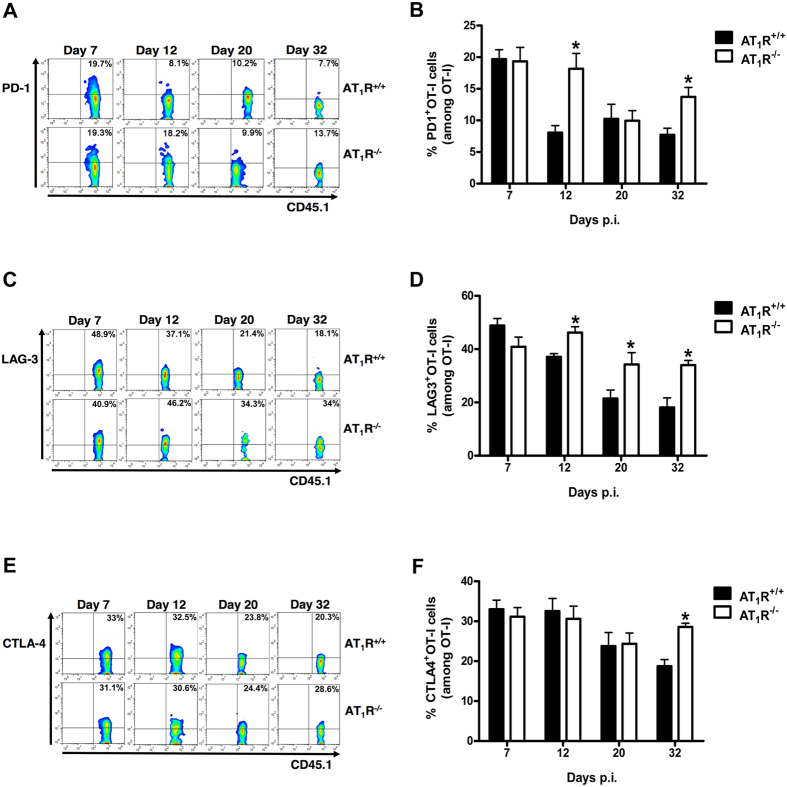
AT_1_R decreases exhaustion in antigen-specific CD8^+^ T cells. Percentage of cells expressing exhaustion markers, PD-1, LAG-3, and CTLA-4, were evaluated in AT_1_R^+/+^ and AT_1_R^−/−^ OT-I cells (CD8^+^CD45.1^+^) isolated from the spleen of immunized recipient mice (CD45.2^+^) at indicated time points post immunization. The gating strategy used for flow cytometry analysis is indicated in the Materials and Methods section. (**A,B**) Representative dot plots and percentage of PD-1^+^ OT-I cells among total OT-I cells in the spleen on days 7 (p = 0.9), 12 (*p = 0.0192), 20 (p = 0.9), and 32 (*p = 0.0285) post immunization. (**C,D**) Representative dot plots and percentage of LAG-3^+^ OT-I cells among total OT-I cells in the spleen on days 7 (p = 0.11), 12 (*p = 0.0024), 20 (*p = 0.0468), and 32 (*p = 0.0074) post immunization. (**E,F**) Representative dot plots and percentage of CTLA-4^+^ OT-I cells among total OT-I cells in the spleen on days 7 (p = 0.6), 12 (p = 0.6907), 20 (p = 0.551), and 32 (*p = 0.0197) post immunization. Data are means ± SEM of 4 mice per group and are representative of 2 independent experiments with similar results for each indicated time point. Abbreviations: CTLA-4, cytotoxic T-lymphocyte-associated protein 4; LAG-3, lymphocyte-activation gene 3; PD-1, programmed cell death 1.

Figure 5 and Supplementary S6A shows that PD1 expression is higher in AT_1_R^−/−^ cells than in AT_1_R^+/+^ cells at day 12 p.i. (effector phase) and day 32 p.i. (memory phase). The frequency of LAG3^+^ cells and expression in AT_1_R^+/+^ OT-I cells decrease after 7 days p.i., whereas in AT_1_R^−/−^ OT-I cells they remain significantly higher from day 12 (Fig. 5C,D and Supplementary Fig. S6B). CTLA4 expression and frequency upregulate in AT_1_R^−/−^ OT-I cells at day 32 (Fig. 5E,F and Supplementary Fig. S6C).

The higher expression of such markers in AT_1_R^−/−^ antigen-specific CD8^+^ T cells is in agreement with the observations described above, supporting the idea of higher activation along with exhaustion of AT_1_R-deficient CD8^+^ T cells, mainly 12 days p.i.

### AT_1_R inhibits the upregulation of IL7-Rα expression in antigen-specific CD8^+^ T cells during the memory development phase

**Figure 6:**
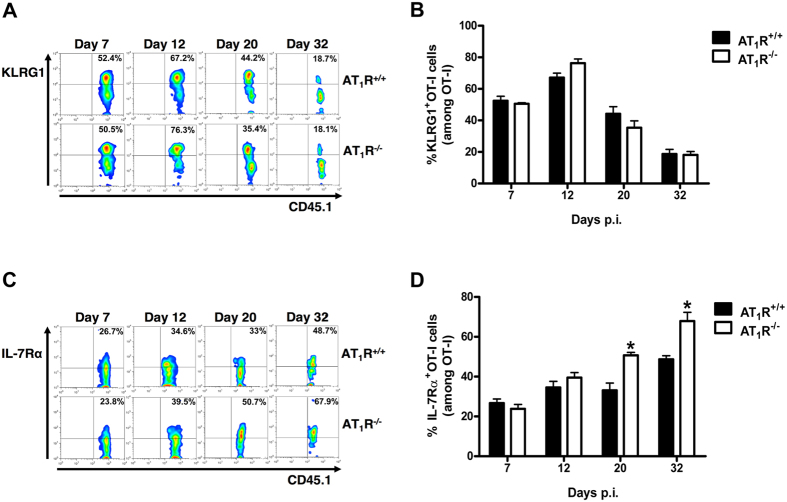
AT_1_R inhibits the upregulation of IL7-Rα expression in antigen-specific CD8^+^ T cells during the memory development phase. Percentage of cells expressing IL-7Rα and KLRG-1 were evaluated in AT_1_R^+/+^ and AT_1_R^−/−^ OT-I cells (CD8^+^CD45.1^+^) harvested from the spleen of immunized recipient mice (CD45.2^+^) and analyzed at the indicated time points post immunization. The gating strategy used for flow cytometry analysis is indicated in the Material and Methods section. (**A,B**) Representative dot plots and percentage of KLRG1^+^ OT-I cells among total OT-I cells in the spleen on days 7 (p = 0.5902), 12 (p = 0.07), 20 (p = 0.2654), and 32 (p = 0.8903) post immunization. (**C,D**) Representative dot plots and percentage of IL-7Rα ^+^ OT-I cells among total OT-I cells in the spleen on days 7 (p = 0.4388), 12 (p = 0.2857), 20 (*p = 0.0089), and 32 (*p = 0.0429) post immunization. Data are means ± SEM of 4 mice per group and are representative of 2 independent experiments with similar results for each indicated day. Abbreviations: IL7-Rα, interleukin-7 receptor α-chain; KLRG-1, killer cell lectin like receptor G1.

Next, we evaluated the expression levels of markers of short-term effector cells and memory cells, KLRG1 and IL-7Rα (CD127), respectively^28^,^30^,^38^,^39^. Figures 6 and S4 shows that during effector response (days 7 and 12), the frequency and expression of KLRG1 and IL-7Rα are similar in both AT_1_R^+/+^ and AT_1_R^−/−^ OT-I cells. As also described previously in another system^38^, from day 12 KLRG1 expression and frequency start to decrease, whereas IL-7Rα expression is increased, which suggests the appearance of terminally differentiated antigen-specific memory cells (Fig. 6 and Supplementary Fig. S6). However, IL-7Rα expression and frequency are significantly higher in AT_1_R^−/−^ OT-I cells (Fig. 6 and Supplementary Fig. S6E). Together with CD44 and CD62L expression, this result suggests that after the contraction, more surviving AT_1_R^−/−^ antigen-specific CD8^+^ T cells transition to a central memory phenotype.

### AT_1_R fosters polyfunctional CD8^+^ T cells during the effector phase and cytotoxic CD8^+^ T cells during the memory phase

Because AT_1_R seems to be important to prevent exacerbated activation and decrease exhaustion, we evaluated the role of AT_1_R in the functional capacity of antigen-specific CD8^+^ T cells. Functional populations of cells with cytotoxic capacity, measured by the surface mobilization of CD107a, as well as producing cytokines (IFN-γ, TNF-α, and IL-2), were determined after *ex vivo* restimulation with SIINFEKL-pulsed target cells (Supplementary Fig. S7)^29^,^42^,^43^.

**Figure 7:**
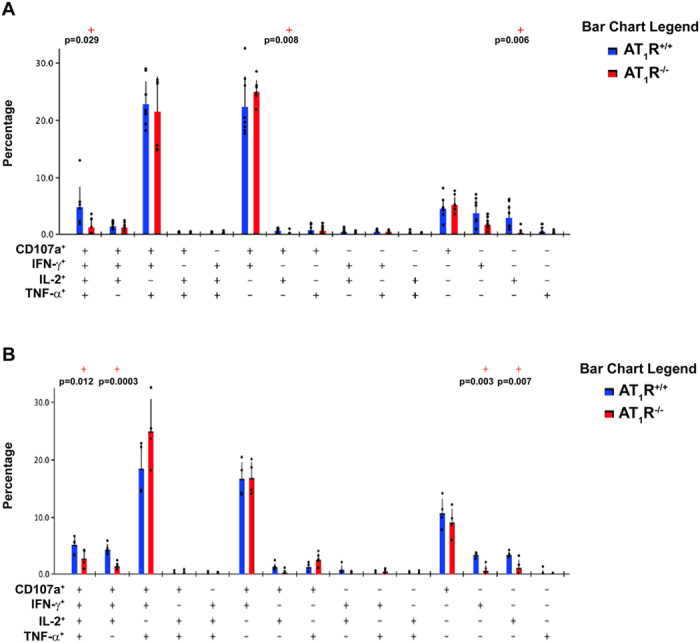
AT_1_R expression in effector antigen-specific CD8^+^ T cells fosters polyfunctional capacity. 1 × 10^4^ naive AT_1_R^+/+^ or AT_1_R^−/−^ CD45.1^+^ OT-I cells were adoptively transferred to WT C57BL/6 (CD45.2^+^) recipients 1 day before intravenous inoculation with 1 × 10^5^ γ-irradiated *P. berghei CS*^*5M*^ sporozoites. (**A,B**) Seven (**A**) or 12 days (B) post immunization, spleen suspensions were stimulated *ex vivo* with SIINFEKL peptide-coated target cells. CD8^+^ T-cell functionality was determined by the combinatorial analysis of the functional subsets of responding CD8^+^CD45.1^+^ T cells residing in the spleen. Responding cells are defined by the capacity to produce at least 1 of the 3 cytokines and/or surface mobilization of CD107a after *ex vivo* stimulation. The bar charts indicate the frequency of CD8^+^CD45.1^+^ T lymphocytes (OT-I cells) expressing each of the 15 possible combinations of CD107a, IFN-γ, TNF-α, and IL- 2 among responding OT-I cells at (**A**) day 7 and (**B**) day 12 post immunization. Bars represent means ± SD of 5 or 6 mice per group and are representative of 2 independent experiments with similar results for each indicated time point.

**Figure 8:**
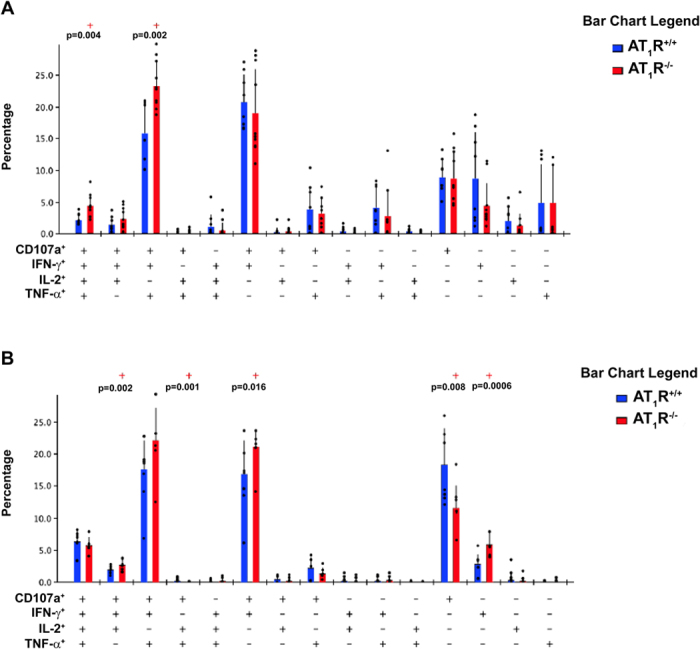
AT_1_R promotes cytotoxic antigen-specific CD8^+^ T cells during the memory phase. 1 × 10^4^ naive AT_1_R^+/+^ or AT_1_R^−/−^ CD45.1^+^ OT-I cells were adoptively transferred to WT C57BL/6 (CD45.2^+^) recipients 1 day before intravenous inoculation with 1 × 10^5^ γ-irradiated *P. berghei CS*^*5M*^ sporozoites. (**A,B**) Twenty (**A**) or 32 days (**B**) post immunization, spleen suspensions were stimulated *ex vivo* with SIINFEKL peptide-coated target cells. CD8^+^ T-cell functionality was determined by the combinatorial analysis of the functional subsets of responding CD8^+^CD45.1^+^ T cells residing in the spleen. Responding cells are defined by the capacity to produce at least 1 of the 3 cytokines and/or surface mobilization of CD107a after *ex vivo* stimulation. The bar charts indicate the frequency of CD8^+^CD45.1^+^ T lymphocytes (OT-I cells) expressing each of the 15 possible combinations of CD107a, IFN-γ, TNF-α, and IL- 2 among responding OT-I cells at days (A) 20 and (B) 32 post immunization. Bars represent mean ± SD of 5 or 6 mice per group and are representative of 3 independent experiments with similar results for each indicated time point.

Boolean gates combinatory analysis at days 7, the peak of the effector WT and AT_1_R^−/−^ OT-I responses, and 12 p.i. revealed a significant percentage of AT_1_R^+/+^ OT-I cells differentiated into polyfunctional effector cells, capable of simultaneously producing 2 or more cytokines and/or with cytotoxic capacity (represented by CD107a expression on the cell surface) (Fig. 7). This functional capacity decreases over time (memory development phase), and a higher percentage of memory cells with monofunctional phenotype, mainly cytotoxic (CD107a^+^), become evident (Fig. 8). Meanwhile, there is a lower frequency of AT_1_R^−/−^ OT-I cells with polyfunctional capacity, mainly showing 4 (CD107a^+^ IFN-γ^+^ TNF-α^+^ IL-2^+^) or 3 (CD107a^+^ IFN-γ^+^ IL-2^+^) functions during the effector phase, as observed at days 7 and 12 p.i. (Fig. 7). Moreover, a lower percentage of monofunctional AT_1_R^−/−^ OT-I cells cells producing only IFN-γ or IL-2 was also observed, in agree with the results described above (Fig. 7).

**Figure 9:**
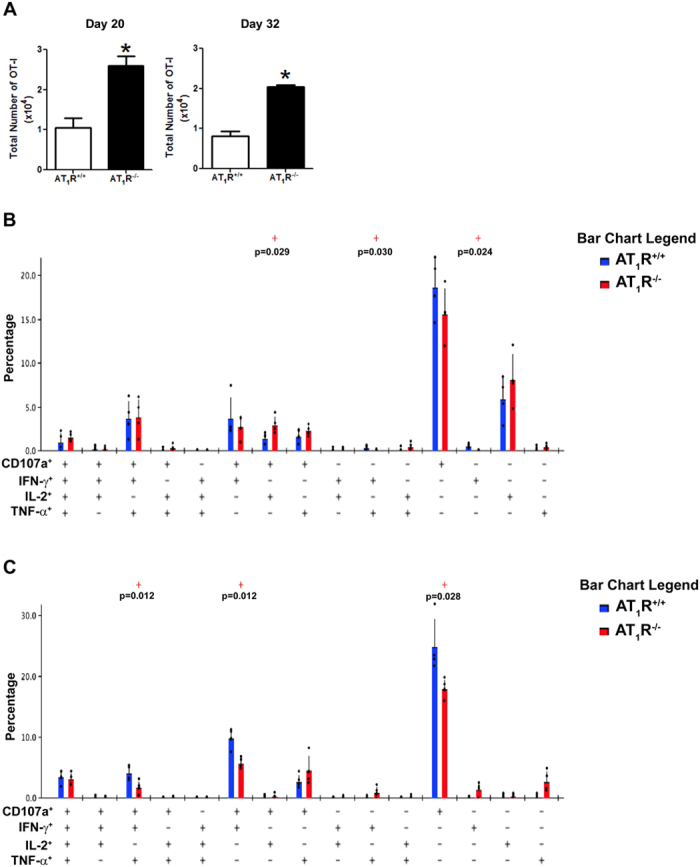
AT_1_R induces cytotoxic memory antigen-specific CD8^+^ T cells in the liver. 1 × 10^4^ naive AT_1_R^+/+^ or AT_1_R^−/−^ CD45.1^+^ OT-I cells were adoptively transferred to WT C57BL/6 (CD45.2^+^) recipients 1 day before intravenous inoculation with 1 × 10^5^ γ-irradiated *P. berghei CS*^*5M*^ sporozoites. (**A**) Total number of AT_1_R^+/+^ or AT_1_R^−/−^ OT-I cells per liver at days 20 (*p = 0.0073) and 32 (*p = 0.0013) post inoculation, calculated as the frequencies obtained by CD8^+^CD45.1^+^ staining, multiplied by the total number of cells obtained after liver excision. Data are means ± SEM of 4 mice per group and are representative of 2 independent experiments with similar results for each indicated time point. (**B,C**) Twenty (B) or 32 (C) days post immunization, T cells were recovered from the liver and were stimulated *ex vivo* with SIINFEKL peptide-coated target cells. CD8^+^ T-cell functionality was determined by the combinatorial analysis of the functional subsets of responding CD8^+^CD45.1^+^ T cells residing in the liver. Responding cells are defined by the capacity to produce at least 1 of the 3 cytokines and/or surface mobilization of CD107a after *ex vivo* stimulation. The bar charts indicate the frequency of CD8^+^CD45.1^+^ T lymphocytes (OT-I cells) expressing each of the 15 possible combinations of CD107a, IFN-γ, TNF-α, and IL- 2 among responding OT-I cells at days (B) 20 and (C) 32 post immunization. Bars represent means ± SD of 4 mice per group and are representative of 2 independent experiments with similar results for each indicated time point.

During the memory phase (at days 20 and 32), there is an increase in the percentage of AT_1_R^−/−^ OT-I cells with polyfunctional capacity (Fig. 8). In addition, there is a decrease in memory AT_1_R^−/−^ OT-I cells showing only cytotoxic function (CD107a expression only) in comparison with AT_1_R^+/+^ OT-I cells at day 32 (Fig. 8). Interestingly, in the liver, a significantly higher number of AT_1_R^–/–^ OT-I cells was observed at 20 and 32 days after immunization (Fig. 9). However, a lower percentage of AT_1_R^−/−^ OT-I cells producing CD107a, alone or in combination, in comparison with AT_1_R^+/+^ OT-I cells was verified at day 32 p.i. (Fig. 9). These results demonstrate that AT_1_R is important to the generation of antigen-specific CD8^+^ T cells with cytotoxic function during the memory phase.

### Lack of AT_1_R expression in parasite-specific CD8^+^ T cells ameliorates protection against lethal disease induced by *P. berghei* ANKA infection in mice

**Figure 10:**
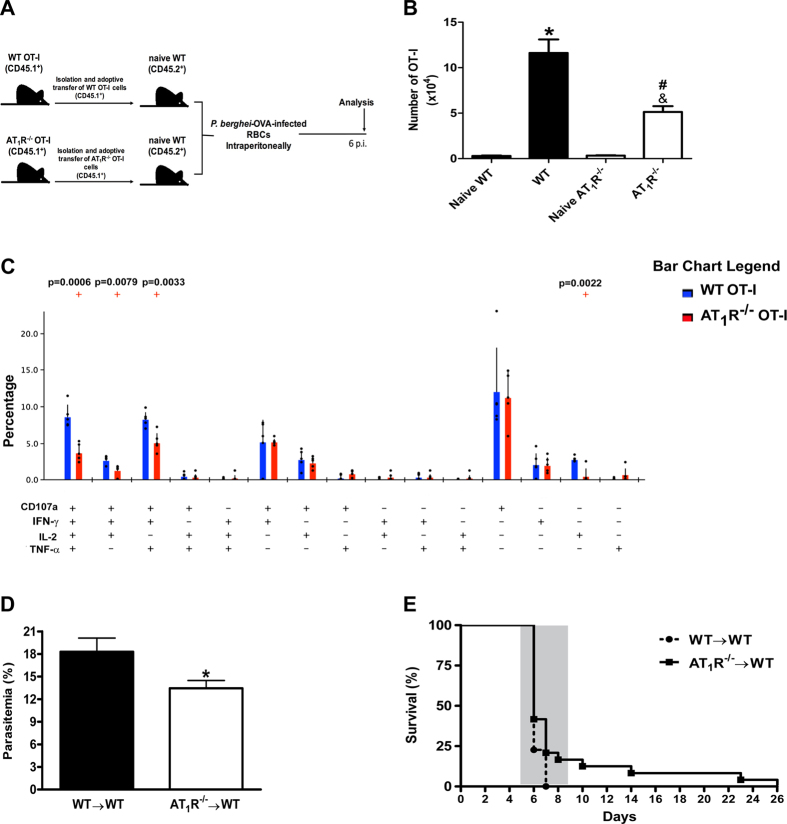
Lack of AT_1_R expression in parasite-specific CD8^+^ T cells protects mice from lethal disease induced by blood-stage PbA infection. 1 × 10^4^ naive WT or AT_1_R^−/−^ CD45.1^+^ OT-I cells were adoptively transferred to WT C57BL/6 (CD45.2^+^) recipients 1 day before infection with 5 × 10^6^ RBCs infected with OVA-PbA. (**A**) Schematics of the experimental design. 1 × 10^4^ Naive WT (AT_1_R^+/+^) or AT_1_R^−/−^ OT-I cells (CD8^+^CD45.1^+^) were adoptively transferred to WT C57BL/6 mice (CD45.2^+^) recipients 1 day before intraperitoneal injection of 5 × 10^6^ infected red blood cells (RBCs) with *P. berghei* ANKA expressing ovalbumin (OVA-PbA). Mice were euthanized at the indicated time point for recovery and analysis of OT-I cells. (**B**) Total number of WT or AT_1_R^−/−^ OT-I cells per spleen at day 6 post infection, calculated as the frequencies obtained by CD8^+^CD45.1^+^ staining, multiplied by the total number of cells obtained after spleen excision. Data are means ± SEM of 5 mice per group and are representative of 3 independent experiments with similar results (*p < 0.0001 in relation to naïve WT OT-I cells; ^&^p < 0.0001 in relation to naïve AT_1_R^−/−^ OT-I cells ^#^p < 0.0001 in relation to WT OT-I cells). (**C**) CD8^+^ T cell functionality was determined by the combinatorial analysis of the functional subsets of responding CD8^+^CD45.1^+^ T cells residing in the spleen. Responding cells are defined by the capacity to produce at least one of the three cytokines and/or surface mobilization of CD107a after *ex vivo* stimulation. The bar charts indicate the frequency of CD8^+^CD45.1^+^ T lymphocytes (OT-I cells) expressing each of the 15 possible combinations of CD107a, IFN-γ, TNF-α, and IL-2 among responding OT-I cells. Bars represent means ± SD of five mice per group and are representative of three independent experiments with similar results. (**D**) Parasitemia was determined by Giemsa-stained blood smears in infected mice that received WT (WT → WT) or AT_1_R^−/−^ OT-I cells (AT_1_R^−/−^ → WT). Data are means ± SEM of five mice per group and are representative of three independent experiments with similar results (*p = 0.03). (**E**) Mortality was checked daily in the mice infected with OVA-PbA that received WT (WT→WT; dotted line) or AT_1_R^−/−^ (AT_1_R^−/−^ → WT; filled line) OT-I cells. The shaded area indicates the time when mice displayed ECM symptoms. After this time, mice died with hyperparasitemia. Data are are representative of 3 independent experiments with similar results with 10 mice per group (*p = 0.0365).

So far our results show that AT_1_R plays a pivotal role in the antigen-specific CD8^+^ T cell response, influencing the expansion, contraction, and expression of IL-2R, IL-7R, and other surface markers. Previously, we showed that pharmacological inhibition of AT_1_R protects mice against cerebral malaria (CM) during blood-stage malaria^13^. Then, to verify the biological significance of the altered CD8^+^ T cell function due to lack of AT_1_R, we examined the lethal disease induced by *P. berghei* ANKA (PbA) infection^13^,^32^,^33^,^44^,^45^. Parasitemia and survival curves of mice that received WT or AT_1_R^−/−^ OT-I cells were evaluated after infection with PbA-expressing OVA (OVA-PbA)^33^ (Fig. 10A). Similar to AT_1_R^−/−^ cells at days 7 and 12 (effector phase) in γ-spz immunized mice (Figs 1 and 7), the expansion of AT_1_R^−/−^ OT-I cells is lower and they are less polyfunctional at day 6 p.i. in OVA-PbA-infected mice (Fig. 10B,C). Interestingly, parasitemia at day 6, when CM signs appear^13^,^32^,^33^,^44^,^45^, was 32% lower in the infected mice that received AT_1_R^−/−^ OT-I cells (AT_1_R^−/−^ → WT) (Fig. 10D). This suggests that OT-I cells lacking AT_1_R have a higher capacity to limit the parasite burden.

It is well known that C57BL/6 mice infected with PbA die because of CM between 6 and 8 days p.i., whereas infected mice dying after that are affected by hyperparasitemia and severe anemia^44^,^45^. We characterized CM by observing several clinical signs in infected mice, such as hemi- or paraplegia, ataxia, deviation of the head, convulsions, and coma between 5 and 8 days p.i.; deaths were checked daily. Importantly, analysis of survival curves revealed that 100% of OVA-PbA-infected mice injected with WT OT-I cells (WT → WT) exhibited CM signs within 5–7 days p.i. and rapidly died of fatal CM (Fig. 10E). Most (80%) died on day 6 p.i., and all animals from this group were dead by day 7 p.i. (Fig. 10E). In contrast, in the infected mice injected with AT_1_R^−/−^ OT-I cells (AT_1_R^−/−^ → WT), neurological signs of CM began to appear later and the mortality rate increased slowly throughout days 6–8. These mice showed a significant amelioration of survival; 40% and 25% of mice survived at days 6 and 7, respectively (Fig. 10E). In the mice that did not develop CM, the mortality rate increased until day 26 and mice died as a result of hyperparasitemia (up to 70%). In agreement with our previous study^13^, these results highlight that altered functions of parasite-specific CD8^+^ T cells due to ablation of AT_1_R expression ameliorates protection of mice against the lethal disease induced by PbA infection.

## Discussion

In this work, we used a model of immunized mice with irradiated *Plasmodium* sporozoites to evaluate the role of AT_1_R in the regulation of the antigen-specific CD8^+^ T-cell response. Although Ang II binds 2 different receptors, AT_1_R and AT_2_R, previous studies have shown that AT_1_R expression is upregulated in T cells *in vitro* and during *Plasmodium berghei* ANKA (PbA) infection and mediates most of the effects of Ang II in T cells^5^,^6^,^7^,^8^,^9^,^10^,^11^,^12^,^13^,^14^,^15^,^18^,^20^,^21^. The upregulated AT_1_R plays a role in activation, effector function, CD8^+^ T-cell sequestration in the brain, cerebral edema, and behavioral impairment during the blood stage of PbA infection, as well as in other models of diseases^10^,^11^,^13^,^15^,^18^,^19^,^20^,^21^. However, the specific role of AT_1_R expressed by antigen-specific CD8^+^ T cells in regulating their response is not yet clear yet. To our knowledge, this is the first study highlighting the influence of this receptor in the course of the antigen-specific CD8^+^ T-cell response.

Many studies have taken advantage of the use of different transgenic lineages of *Plasmodium*^32^,^33^,^34^. For instance, *P. berghei CS*^5M^ is a *P. berghei* strain in which the native H-2K^d^–restricted epitope SYPSAEKI from the C-terminus of the CS protein is substituted with the H-2K^b^–restricted epitope SIINFEKL^34^. In this parasite lineage, the SIINFEKL is presented as a natural CS epitope, including a stage-specific pattern of expression, antigen presentation, and processing^34^. In addition, only in C57BL/6 mice immunized with *P. berghei* CS^5M^ parasites there is a robust SIINFEKL-specific immune response, differently in the mice immunized with the parental lineage *P. berghei* ANKA^34^. Moreover, upregulation of CD69 and the production of IFN-γ by OT-I cells occurs only in recipient mice injected with *P. berghei* CS^5M^ sporozoites^36^. Here we also observed CD69 upregulation, as well as other markers of activation, only in the cells that respond to the SIINFEKL epitope. The *P. berghei* CS^5M^ sporozoites are not able to induce activation of endogenous CD8^+^ T-cell clones specific to other antigens. Recently, it was demonstrated that effector antigen-specific CD8^+^ T cells activity is restricted to the immediate microenvironment of the infected cell following interactions with the cognate antigen and bystander killing of parasites was not observed^37^. Together, these observations explicitly demonstrate that, during γ-spz immunization, antigen-specific CD8^+^ T cells are stimulated and respond only to the cognate antigen in an antigen specific-manner.

It is known that the CD8^+^ T-cell response initiates by antigen recognition by naive cells, upregulation of activation molecules, and clonal expansion, followed by acquisition of effector functions^22^,^24^,^25^. In this work, a peak in the number of OT-I cells in the spleen was observed at 7 days p.i., which suddenly decreased due to homeostatic contraction, in accordance with previous studies using this model and in other types of infections^25^,^26^,^39^,^46^,^47^. In addition, our observations suggest that during the effector phase, AT_1_R^−/−^ CD8^+^ T cells have a lower rate of proliferation but also contract to lesser extension. Since IL-2 signaling is important for CD8^+^ T-cell proliferation^24^,^29^,^30^,^31^,^32^, a possible molecular mechanism is that AT_1_R upregulates IL-2R and stimulates IL-2 production by CD8^+^ T cells during the effector phase. In agree, previous studies demonstrated that Ang II/AT_1_R axis promotes T-cell proliferation by increasing IL-2 production, via induction of NADPH oxidase-mediated ROS generation and/or activation of calcineurin phosphatase^5^,^6^,^7^,^10^. After reaching a peak of expansion, the CSP-specific CD8^+^ T cells reduce extensively due to massive apoptosis, becoming a stable memory population capable of mediating protection against a posterior sporozoite challenge^24^,^25^,^26^,^38^,^39^. Our data suggest that AT_1_R accelerates contraction of the responding population, which is confirmed by the higher inclination in the curve after the peak of clonal expansion at day 7. A possible mechanism is the lower expression of IL-7R in the AT_1_R^+/+^ cells during the contraction phase, because IL-7 plays an overlapping and complimentary role with IL-2 in the survival of memory cell precursors during memory cell development^28^,^30^,^48^,^49^,^50^. Given that AT_1_R-deficient cells, expressing higher levels of IL-7R, survive to generate a similar level of memory as AT_1_R-sufficient cells at day 32 pi in the spleen. In the liver, effector cells lacking AT_1_R may also have an intrinsic survival advantage because they generate a higher number of memory cells. This advantage is particularly critical for memory cells in the liver, where there may be a shortage of the cytokine or chemokine interactions required to sustain the formation of memory cells^35^. Future experiments will address the exact molecular mechanisms involved in this phenomenon.

AT_1_R^−/−^ OT-I cells also significantly express higher levels of CD69 and CD160, and less of CD62L during effector phase, which indicates that AT_1_R decreases the exacerbated activation of antigen-specific CD8^+^ T cells. During the memory development phase (days 20 and 32), CD62L and IL-7Rα expression were higher in AT_1_R^−/−^ OT-I cells, which indicate a faster transition of AT_1_R^−/−^ antigen-specific CD8^+^ T cells to a central memory phenotype^28^,^30^,^38^,^39^,^48^,^49^,^50^. The higher expression of CD69 and CD44 in memory AT_1_R^−/−^ OT-I cells suggest that these cells are continuing to respond to antigen, which could contribute to their exhausted phenotype. Another possible impact is the increase of IL-2 production by memory AT_1_R^−/−^ OT-I cells. CD69 signaling through NFAT and AP-1 transcription factors upregulates IL-2 expression^51^.

Besides higher activation, we observed a higher percentage of AT_1_R-deficient OT-I cells expressing the exhaustion molecules PD-1, CTLA-4, and LAG-3. Typically, exhausted T cells lose their polyfuncitonality, the ability to simultaneously produce cytokines such as IL-2, IFN-γ, TNF-α and to degranulate^52^. This correlates with the lower polyfunctional capacity of AT_1_R^−/−^OT-I cells during the effector phase. Moreover, exhausted CD8^+^ T cells are eventually deleted, which could also explain why AT_1_R^−/−^ cells are present at lower numbers during the effector phase. During memory phase, even though AT_1_R^−/−^ cells are more exaushted, their polyfunctional capacity increases. However, accordingly with the progressive loss of effector functions, the ability to produce IL-2 is lost followed by the ability to produce TNF-α, and then IFN-γ^52^, the percentages of AT_1_R^−/−^ OT-I cells able to produce all cytokines but IL-2 and/or TNF-α are higher when compared to WT cells (Fig. 8).

Here, it was also observed that AT_1_R^–/–^ OT-I cells form a **2.5** larger memory cell population in the liver. This can deliver important information in this model. Parasite elimination is dependent on each and every parasite being found and eliminated by at least one cognate CD8^+^ T cell^37^. In addition, because there are redundant effector mechanisms for parasite elimination (such as IFN-γ and TNF-α secretion), the total number of antigen-specific CD8^+^ T cells is a critical factor in determining the ability of CD8^+^ T cells to control liver-stage malaria parasites^29^,^37^,^53^,^54^,^55^,^56^,^57^. Here, we observed that there were no distinguishable differences in the capacity to produce cytokines between memory WT and AT_1_R^–/–^ OT-I cells in the liver (Supplementary Fig. S7). Although liver-residing AT_1_R^–/–^ OT-I cells are less committed to cytotoxic function, as shown by the polyfunctional analysis, there is not much difference in the overall functionality in comparison with the WT control (Fig. 9). Thus, the number of memory cells becomes an important factor to limit parasite development in the liver^29^,^37^,^56^,^57^. Collectively, these data suggest that it is plausible that AT_1_R^–/–^ memory cells could confer better protection from challenges with live sporozoites. Here we verified that genetic ablation of AT_1_R expression, specifically in parasite-specific CD8^+^ T cells, delays CM-specific neurological signs and confers better protection against PbA-induced lethal disease in mice. In addition, although expansion and the polyfunctional capacity of effector AT_1_R^−/−^ OT-I cells are also lower during the blood stage, they showed a better capacity to limit parasitemia when CM signs appeared (day 6 p.i.). This is in agreement with evidence showing that higher numbers of CD8^+^ T cells and production of IFN-γ drive the rapid increase in parasite biomass from day 6 p.i., when mice develop ECM^58^,^59^. Moreover, these effects have an important biological significance, because an adjuvant treatment that delays mortality is clinically relevant for human malaria, since most patients with CM die before the beneficial effects of conventional anti-malarial treatment are observed^60^. Thus, these data corroborate that modulation of the Ang II/AT_1_R axis could be an interesting target in an adjunctive therapy to improve poor malaria outcomes^13^,^61^.

Together, our results indicate that AT_1_R plays a pivotal role in the antigen-specific CD8^+^ T cell response, influencing the proliferation and expression of IL-2R, IL-7R, and other surface markers. Our group and others have shown that Ang II plays a pivotal role in regulating the T cell response in an autocrine or paracrine manner via AT_1_R, which is upregulated in activated T cells^10^,^11^,^12^,^13^,^14^,^15^,^21^. In addition, other studies show that different innate immune cells, such as monocytes, macrophages, and dendritic cells, produce Ang II and express higher levels of mRNA of the different renin-angiotensin system (RAS) components than T cells^8^,^10^,^16^,^17^,^18^,^19^,^20^. During the encounter with the antigen, in the context of co-stimulatory stimuli, Ang II activates AT_1_R in T cells initiating distinct transcriptional profiles that regulate avidity by antigen, activation, clonal expansion, migration and effector function^10^,^11^,^12^,^13^,^14^,^15^,^16^,^17^,^18^,^19^,^20^,^21^. Moreover, because the events that control the initial activation of T cells ultimately determine the fate of the responding cell^30^,^48^, contraction of the effector population and memory development could also be affected by Ang II-induced AT_1_R activation. However, the AT_1_R-induced signaling pathways behind these processes remain undefined. It is known that AT_1_R activates multiple downstream signals, including PKC, MAPKs (ERK1/2, P38 MAPK, JNK), PI3K/PKB/mTOR, tyrosine kinases (Pyk2, c-Src family kinases, Tyk2, FAK), NFκB, Src-JAK/STAT, and NADPH oxidase, a major source of reactive oxygen species (ROS)^62^. Many of these pathways are important to induce pro-inflammatory transcription factors, T cell activation, proliferation, chemotaxis, cytokine production, and to regulate the development of memory CD8^+^ T cells^39^,^49^,^62^,^63^,^64^. Thus, the diversity of the signaling pathways induced by AT_1_R could explain the pivotal role of this receptor in different aspects of T cell response. Thus, blockade of AT_1_R in antigen-specific CD8^+^ T cells could affect a variety of downstream functions and other regulatory signals that shape the kinetics of the T cell response^30^, making AT_1_R-deficient OT-I cells differ from WT OT-1 cells in many aspects. Future experiments will address the role of the Ang II/AT_1_R axis in the function of other immune cells involved in the regulation of the CD8^+^ T-cell response, such as dendritic cells, CD4^+^ T cells, and NK cells, to better understand these different immune modulatory effects of AT_1_R. These studies could be useful in the development of therapeutic strategies aimed at improving the protective memory CD8^+^ T cells or to inhibit harmful effector CD8^+^ T-cell responses.

## Material and Methods

### Mice

Six- to eight-week-old male C57BL/6 mice were used in all experiments. Wild-type (WT) C57BL/6 mice (background CD45.2) were purchased from NCI. OT-I transgenic C57BL/6 mice (background CD45.1) that recognize SIINFEKL (peptide at position 257–264 of chicken ovalbumin OVA) presented by H-2K^b^ were a gift from Dr. David Sacks (National Institute of Allergy and Infectious Disease, Bethesda, MD). AT_1_R^−/−^ mice (B6.129P2-Agtr1^atm1Unc^/J; backcrossed to C57BL/6 at least seven generations; background CD45.2) were purchased from Jackson Laboratories. All mice were housed, bred, and maintained in the animal care facility at Johns Hopkins University. All animal procedures were approved by the Institutional Animal Care and Use Committee (IACUC) of Johns Hopkins University following the National Institutes of Health (NIH) guidelines for animal housing and care.

### Mice genotyping and phenotyping

Mice that had been previously backcrossed to the CD45.1 C57BL/6 background for more than ten generations were used from our colony, as previously described^35^,^64^. C57BL/6-*Agtr1a*^*tm1Unc*^ (CD45.1) were crossed to OT-I transgenic C57BL/6 mice (CD45.1) from our colony and F1 progeny positive for the TCR transgene were crossed back to C57BL/6-*Agtr1a*^*tm1Unc*^ mice (CD45.1) to obtain TCR transgenic mice homozygous for the *Agtr1a*^*tm1Unc*^ targeted mutation. To phenotype the mice carrying transgenic TCR (OT-I), a drop of blood was collected from the tail vein and verified by flow cytometry using anti-mouse Vα2-fluorescein isothiocyanates (FITC), anti-mouse CD45.1-PE, and anti-mouse CD8-APC antibodies (Supplementary Fig. S1A). For genotyping, genomic DNA was extracted from tail clippings using REDExtract-N-Amp tissue PCR kits (Sigma) following the manufacturer’s protocol. Primer information and PCR conditions were obtained from the website of Jackson Laboratory (http://jax.org). For PCR, primer sequences were: WT locus (oIMR0738), TGAGAACACCAATATCACTG; common (oIMR0739), TTCGTAGACAGGCTTGAG; and mutant locus (oIMR6218), CCTTCTATCGCCTTCTTGACG. The PCR yielded products of 520 bp if mutant, 483 bp if WT or both sizes if heterozygote. PCR was performed with the following cycle settings: 94 °C for 3 min; 94 °C for 30 s; 55 °C for 30 s for annealing, and 72 °C for 1 min for elongation (total of 40 cycles).

### Purification of antigen-specific CD8^+^ T cells and adoptive transfer

Single-cell suspensions from spleens of AT_1_R^+/+^ OT-I and AT_1_R^−/−^ OT-I mice were obtained by grinding the spleen between the ground ends of 2 microscope slides and then filtering through 100-μm pore size nylon mesh. CD8^+^ T cells were purified by negative selection using magnetic beads following the manufacturer’s protocol (CD8a^+^ T-cell isolation kit; Miltenyi Biotech). Isolated cells were stained with APC-conjugated anti-CD8, FITC-conjugated Vα2, and PE-conjugated CD45.1, and the purity was evaluated by FACS analysis. We obtained approximately 90% of CD8^+^ Vα2^+^ CD45.1^+^ T-lymphocyte (OT-I cells) enrichment (Supplementary Fig. S1B). Adoptive transfer was performed by intravenous injection of 1 × 10^4^ naive AT_1_R^+/+^ or AT_1_R^−/−^ OT-I cells (CD45.1^+^) into recipient WT C57BL/6 mice (CD45.2^+^) 1 day before sporozoite inoculation (Fig. 1A).

### Parasites and immunization

*P. berghei CS*^*5M*^ parasite (carrying the H2-K^b^ SIINFEKL epitope) was generated as previously described^36^. *P. berghei CS*^*5M*^ spz were harvested from the salivary glands of infected female *Anopheles stephensi* mosquitoes and were radiation attenuated in a cesium radiator at 20,000 rad. Mice that received naive AT_1_R^+/+^ or AT_1_R^−/−^ OT-I cells were immunized with intravenous injection of 1 × 10^5^ γ-spz, as previously described^21^,^36^,^37^. Mice were euthanized 3 days p.i. to evaluate early cellular proliferation and related mechanisms, and at days 7, 12, 20, and 32 to evaluate expansion/contraction, differentiation, and function of antigen-specific CD8^+^ cells during the effector (days 7 and 12) and memory phases (days 20 and 32) (Fig. 1A).

The transgenic *Plasmodium berghei* ANKA (OVA-PbA) with truncated C-terminal fragment of OVA (amino acids 150–386) fused to the N-terminal sequence (amino acids 1–5) of the PbA heat shock protein (hsp) 70 gene was also used^33^. A cryopreserved sample of transgenic *Plasmodium berghei* ANKA-infected red blood cells (RBCs) was kindly provided by Dr. Katsuyuki Yui, Nagasaki University, Nagasaki, Japan. The sample was thawed and inoculated intraperitoneally into a naive C57BL/6 mouse. Cells were maintained in mice up to seven passages before use. Mice that received naive AT_1_R^+/+^ or AT_1_R^−/−^ OT-I cells were infected with OVA-PbA by intraperitoneal injection of 5 × 10^6^ infected RBCs. The condition of the mice was checked daily. Parasitemia was monitored by microscopic examination of standard blood films at day 6 p.i., at the onset of signs of CM^13^,^32^,^33^.

### Lymphocyte isolation

The spleens were harvested on days 3–32 and the liver on days 20 and 32 after immunization as indicated. Single-cell suspensions of lymphocytes from these organs were obtained by grinding the tissues between the ground ends of 2 microscope slides and then filtering them through 100-μm pore size nylon mesh. Numbers of OT-I cells were estimated by automated cell counting using the Trypan blue dye exclusion method (viability was found to be higher than 95%) and flow cytometry with anti-CD45.1 and anti-CD8 antibodies.

Livers were first perfused with 10 ml of ice-cold Hank’s balanced salt solution through the hepatic portal system. To isolate intrahepatic lymphocytes, the liver pellet was resuspended in a 35% Percoll gradient (GE Healthcare), followed by centrifugation at 500 × *g* for 20 min at room temperature. Peripheral blood mononuclear cells were purified through a density gradient (Lymphocyte Separation Medium; Mediatech) at 1250 × *g* for 20 min at room temperature without braking. All lymphocytes were prepared and resuspended in DMEM supplemented with 10% heat-inactivated fetal bovine serum (FBS), 50 mM sodium bicarbonate, 2 mM glutamine, 100 U/ml penicillin, 100 μg/ml streptomycin, 25 mM HEPES.

### Quantification of CD8^+^ T-cell expansion *in vivo*

For evaluation of cellular expansion, expansion of OT-1 cells was measured by flow cytometry and cell count on days 3, 7, 12, 20, and 32 p.i. In other experiments, freshly isolated AT_1_R^+/+^ or AT_1_R^−/−^ OT-I cells were labeled with 1 μM CFSE (Invitrogen) transferred intravenously to recipient C57BL/6 mice, which were immunized with *P. berghei CS*^*5M*^ γ-spz 24 h later. OT-I cells were harvested from the spleen of recipient mice 72 h after immunization, and CFSE dilution was evaluated by flow cytometry (Fig. 2A).

### Antibodies and flow cytometry

All fluorochrome-conjugated monoclonal anti-mouse antibodies were purchased from eBioscience or BD unless stated otherwise: CD45.1 (clone A20); PE-Cy7-conjugated CD8 (clone 53–6.7), CD25 (clone PC61.5), CD122 (clone 5H4) CD69 (H1.2F3), CD160 (clone CNX46–3), CD44 (clone IM7), CD62L (clone MEL-14), KLRG1 (clone 2F1), IL-7Rα (clone A7R34), PD-1 (clone J43), LAG-3 (clone C9B7W), CTLA-4 (clone UC10–4B9), IFN-γ (clone XMG1.2), IL-2 (clone JES6–5H4), TNF-α (clone MP6-XT22), and CD107a (clone 1D4B). PerCP/PE/FITC-conjugated IgG1 and IgG2 isotype controls were all purchased from BD Pharmingen. All results were collected with CellQuest software on a FACSCalibur (Becton Dickinson), and multi-cytokine experiments were performed on a LSRII flow cytometer (Becton Dickinson).

For flow cytometry, isolated AT_1_R^+/+^ or AT_1_R^−/−^ OT-I cells from recipient C57BL/6 mice were incubated with Fc block (anti-mouse CD16 and CD32 antibodies) to block non-specific binding sites for 30 min at 4 °C. Later, the cells were washed and incubated with the appropriate concentration of antibodies cited above. IgG isotypes were used as irrelevant antibodies to define positive populations as indicated in the gate strategy (Supplementary Fig. S1B). At least 10^5^ cells per sample were acquired. Analysis of surface cell markers and intracellular cytokines was performed using FlowJo software (TreeStar). All data were collected and presented in a log scale of fluorescence intensity and presented as plots. The percentage of OT-I cells was determined in a gate of CD8^+^ CD45.1^+^ cells and each analysis was made in relation to the total OT-I cells (gated on CD8^+^ CD45.1^+^ cells). as showed in the gate strategy (Supplementary Fig. S1C). The MFI data were determined by calculating the median fluorescence intensity in the total OT-I cells (gated on CD8^+^ CD45.1^+^ cells) considering the fluorescence of the isotype control, using FlowJo software (TreeStar) (Supplementary Fig. S1C).

### *Ex vivo* stimulation and intracellular staining

El4 cells (T-cell lymphoma cell line of C57BL/6 (H-2^b^) origin) were pulsed with SIINFEKL peptide (10 μg/ml), and control El4 cells (non-pulsed) were incubated at 37 °C for 1 h. Lymphocytes harvested from the spleen of γ-spz immunized mice at days 7, 12, 20, or 32 p.i. were co-cultured with El4 cells pulsed or not for 4 h at 37 °C in the presence of 1:400 brefeldin A (GolgiPlug; BD Bioscience), 1:600 monensin (GolgiStop; BD Bioscience), and anti-CD107a-FITC, a marker of cytotoxic activity. Cells were then permeabilized and fixed using a Cytofix/Cytoperm kit (BD Biosciences) according to the manufacturer’s instructions and stained for intracellular cytokines using anti-IFN-γ-PE-Cy7, anti-TNF-α-Pacific Blue, and anti-IL-2-APC (clone JES6–5H4) at pre-determined concentrations. After staining, cells were washed, diluted and analyzed on a LSR II flow cytometer (BD Bioscience). Functional characterization is based on the capacity of these cells to simultaneously produce or not different cytokines and also express CD107a on the cell surface, which indicates cytotoxic activity. Polyfunctional analysis was done by Boolean combination of gates with FlowJo software, and the data were exported to PESTLE and SPICE software for analysis.

### Data analysis

Each experiment was carried out using 4–6 animals per group (transferred mice with AT_1_R^+/+^ OT-I cells or AT_1_R^−/−^ OT-I cells) for days 3, 7, 12, 20, and 32 p.i. Data are reported as the means ± SEM of 2–3 representative and independent experiments with similar results. Data normality was checked by the Shapiro-Wilk test. Then, for normal distributions, differences between the two groups were compared by a two-tailed Student’s t test or Mann-Whitney test for non-normal distributions, using Prism 5 software (GraphPad Software, version 5). The log-rank (Mantel–Cox) test was used to compare the percentage survival. The level of significance was set at α = 0.05. Permutation tests of significance of polyfunctional distributions were done using SPICE software.

## Additional Information

**How to cite this article**: Silva-Filho, J. L. *et al*. Angiotensin II type-1 receptor (AT_1_R) regulates expansion, differentiation, and functional capacity of antigen-specific CD8^+^ T cells. *Sci. Rep.* **6**, 35997; doi: 10.1038/srep35997 (2016).

**Publisher’s note:** Springer Nature remains neutral with regard to jurisdictional claims in published maps and institutional affiliations.

## Supplementary Material

Supplementary Information
