# Supplementary material for: Angiotensin II type-1 receptor (AT1R) regulates expansion, differentiation, and functional capacity of antigen-specific CD8+ T cells
Source: Sci Rep. 2016 Oct 26;6:35997. doi: 10.1038/srep35997 (PMC5080615; doi:10.1038/srep35997)
Supplement: Supplementary Information [file srep35997-s1.docx]

Angiotensin II type-1 receptor (AT_1_R) regulates expansion, differentiation, and functional capacity of antigen-specific CD8^+^ T cells

João Luiz Silva-Filho, Celso Caruso-Neves, Ana Acacia Sá Pinheiro

**
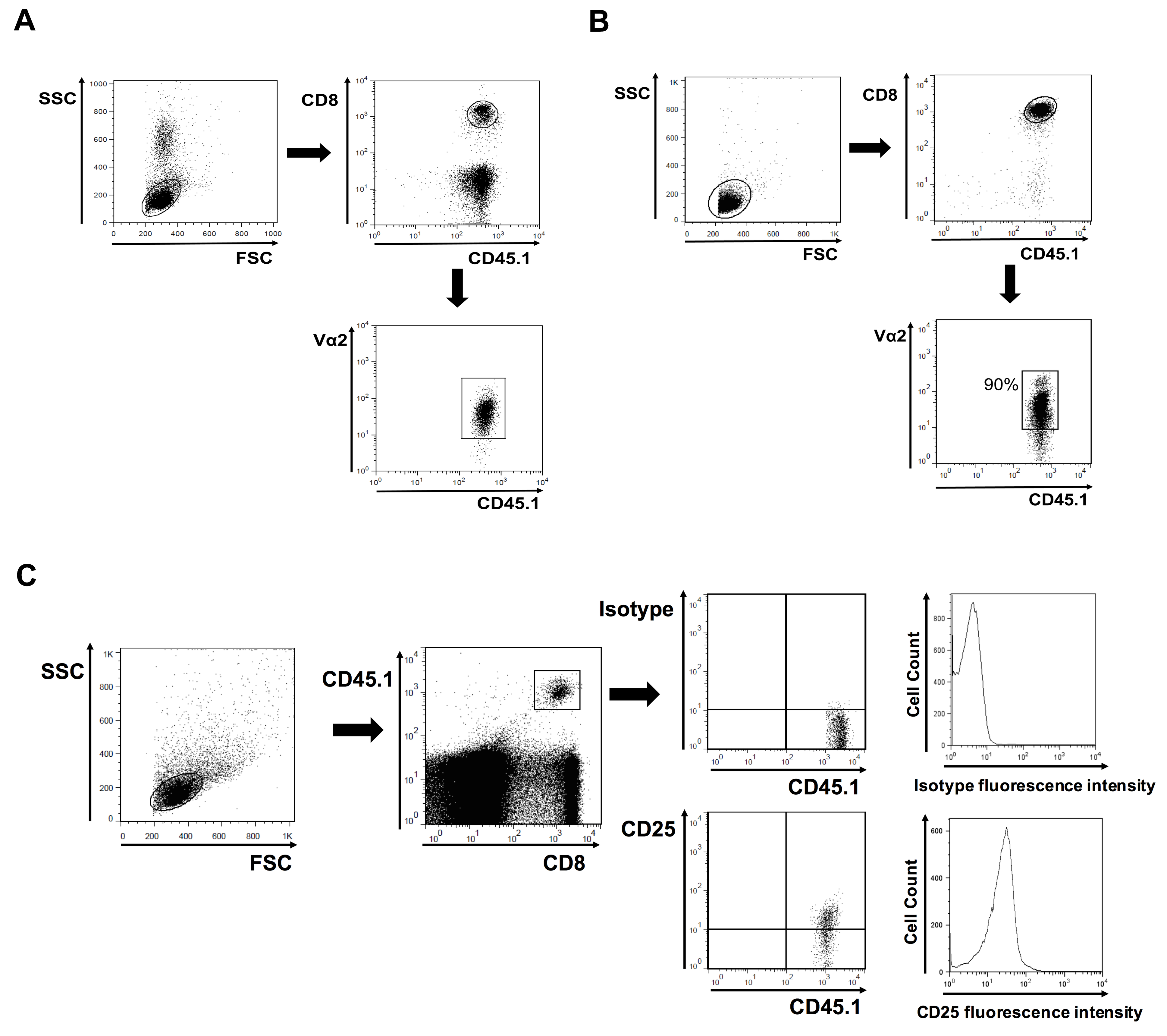
**

**Supplementary Figure S1. Schematics of SIINFEKL-specific CD8^+^ T-cell isolation purity and gating strategy.**

1. To phenotype the mice carrying the transgenic TCR (OT-I), a drop of blood was collected from the tail vein and verified by flow cytometry using anti-mouse Vα2, anti-mouse CD45.1 and anti-mouse CD8 antibodies.
2. To perform adoptive transfer in all experiments, single-cell suspensions from spleens of AT_1_R^+/+^ OT-I and AT_1_R^−/−^ OT-I mice (CD45.1^+^) were obtained, and CD8^+^ T cells were purified by negative selection using magnetic beads as indicated in the Materials and Methods section. SIINFEKL-specific CD8^+^ T cells (OT-I cells) express TCR Vα2-chain. Thus, isolated cells were stained with fluorochrome-conjugated antibodies for CD8, Vα2, and CD45.1. Purity of CD8^+^Vα2^+^CD45.1^+^ T lymphocytes was evaluated by FACS analysis.
   (C) Gating strategy used for flow cytometry analysis. A lymphocyte gate (R1) was defined based on the cells’ forward scatter (FSC) and side scatter (SSC), further gated on CD8^+^CD45.1^+^ lymphocytes (R2), and analyzed for the percentage and levels of expression of different proteins, using corresponding isotypes as irrelevant antibodies to define positive populations.


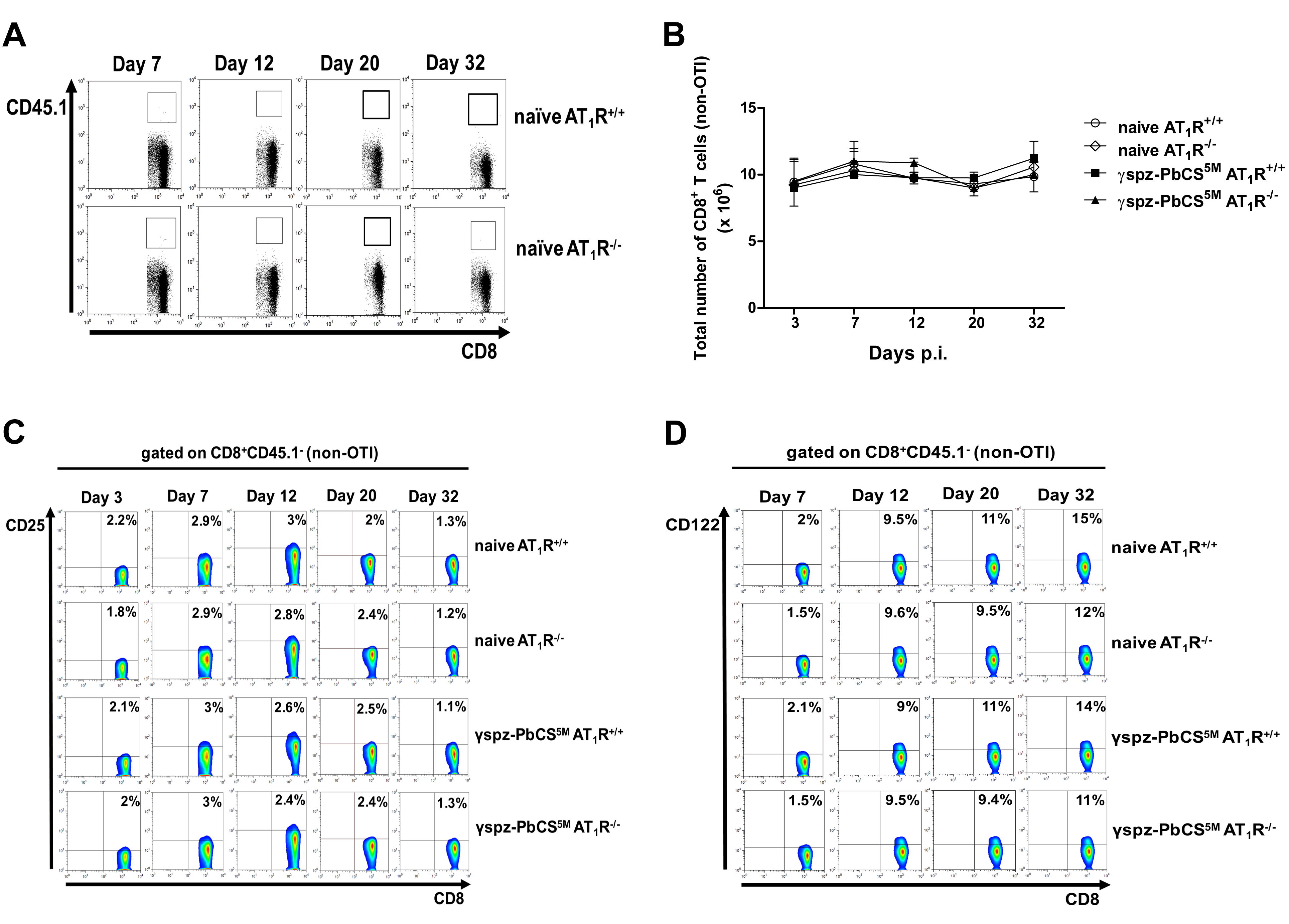


**Supplementary Figure S2. Immunization induces clonal expansion only in the presence of the antigen and changes in CD25 and CD122 expression in CD8^+^ T cells occurs in an antigen specific-manner.**

AT_1_R^+/+^ or AT_1_R^−/−^ OT-I cells (CD8^+^CD45.1^+^) were recovered from the spleen of non-immunized (naïve) recipient mice (CD45.2^+^) and were analyzed in parallel to the cells recovered from immunized mice at days 0, 3, 7, 12, 20, and 32.

1. Representative CD8^+^CD45.1^+^ (OT-I cells) plots gated on total lymphocytes. The gating strategy used for flow cytometry analysis is indicated in the Materials and Methods section.
2. Total number of non-OT-I CD8^+^ T cells (gated on CD8^+^CD45.1^-^ cells) in in the spleen of AT_1_R^+/+^ or AT_1_R^-/-^ OT-I recipient mice (naïve or immunized with γ-spz of *P. berghei* CS^5M^) on days 3, 7, 12, 20 and 32 after immunization.

Data are means ± SEM of 4 mice per group and are representative of 3 independent experiments with similar results for each indicated time point.

(C, D) Representative dot plots of endogenous (non-OT-I; gated on CD8^+^CD45.1^-^ cells) CD25^+^ (C) and CD122^+^CD8^+^ T cells (D) in the spleen of AT_1_R^+/+^ or AT_1_R^-/-^ OT-I recipient mice (naïve or immunized with γ-spz of *P. berghei* CS^5M^) on days 3, 7, 12, 20 and 32 after immunization. Data are means of 4 mice per group and are pooled from 3 independent experiments with similar results for each indicated day.


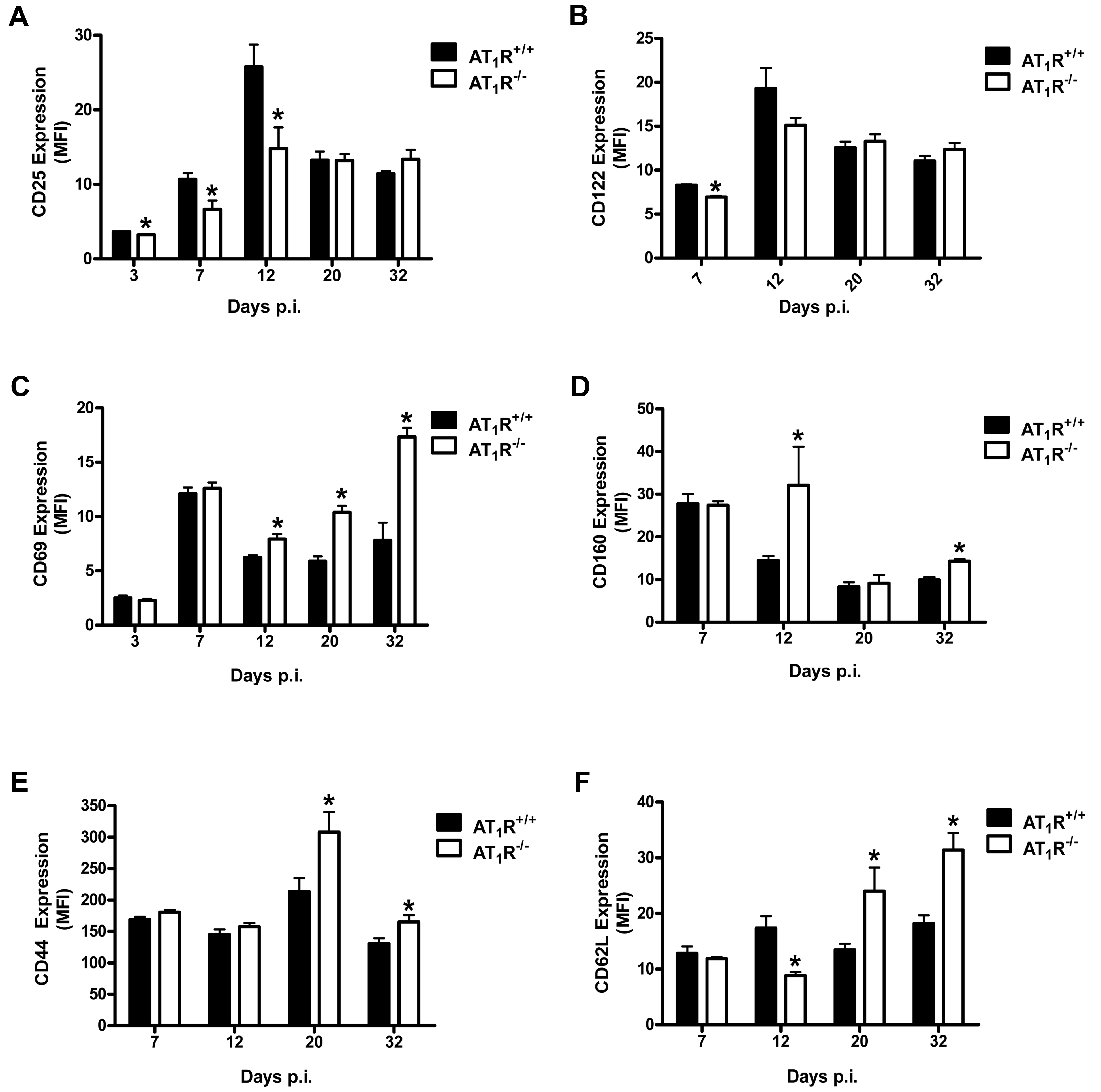


**Supplementary Figure S3. Expression of of IL-2R subunits and activation markers in antigen-specific CD8^+^ T cells.**

1 x 10^4^ naive AT_1_R^+/+^ or AT_1_R^−/−^ CD45.1^+^ OT-I cells were adoptively transferred to WT C57BL/6 (CD45.2^+^) recipients 1 day before intravenous inoculation with 1 × 10^5^ γ-irradiated *P. berghei CS^5M^* sporozoites. The expression of IL-2R subunits CD25 (α-chain) and CD122 (β-chain) and activation markers were evaluated, based on MFI analysis, gated in AT_1_R^+/+^ and AT_1_R^−/−^ OT-I cells (CD8^+^CD45.1^+^) recovered from the spleen of immunized recipient mice (CD45.2^+^) at indicated time points post immunization.

1. Expression levels of CD25 in OT-I cells on days 3 (*p = 0.0037), 7 (*p = 0.0303), 12 (*p = 0.044), 20 (p = 0.9772), and 32 (p = 0.2625) post immunization.
   (B) Expression levels of CD122 in OT-I cells on days 7 (*p = 0.0155), 12 (p = 0.2), 20 (p = 0.572), and 32 (p = 0.2695) post immunization.
   (C) CD69 (day 7, p = 0.5514; day 12, *p = 0.0302; day 20,*p = 0.0139; day 32, *p = 0.0016).
   (D) CD160 (day 7, p = 0.8823; day 12, *p = 0.0291; day 20, p = 0.6792; day 32, *p = 0.0286). Data are means ± SEM of 4 mice per group and are pooled from 2 independent experiments with similar results for each indicated day.
   (E) CD44 (day 7, p = 0.0901; day 12, p = 0.2606; day 20, *p = 0.0254; day 32, *p = 0.0286).
   (F) CD62L (day 7, p = 0.4823; day 12, *p = 0.0186; day 20, *p = 0.0145; day32, *p = 0.0081). Data are means ± SEM of 4 mice per group and are pooled from 3 independent experiments with similar results for each indicated day.

Abbreviations: IL-2R, interleukin-2 receptor; MFI, median of fluorescence intensity.


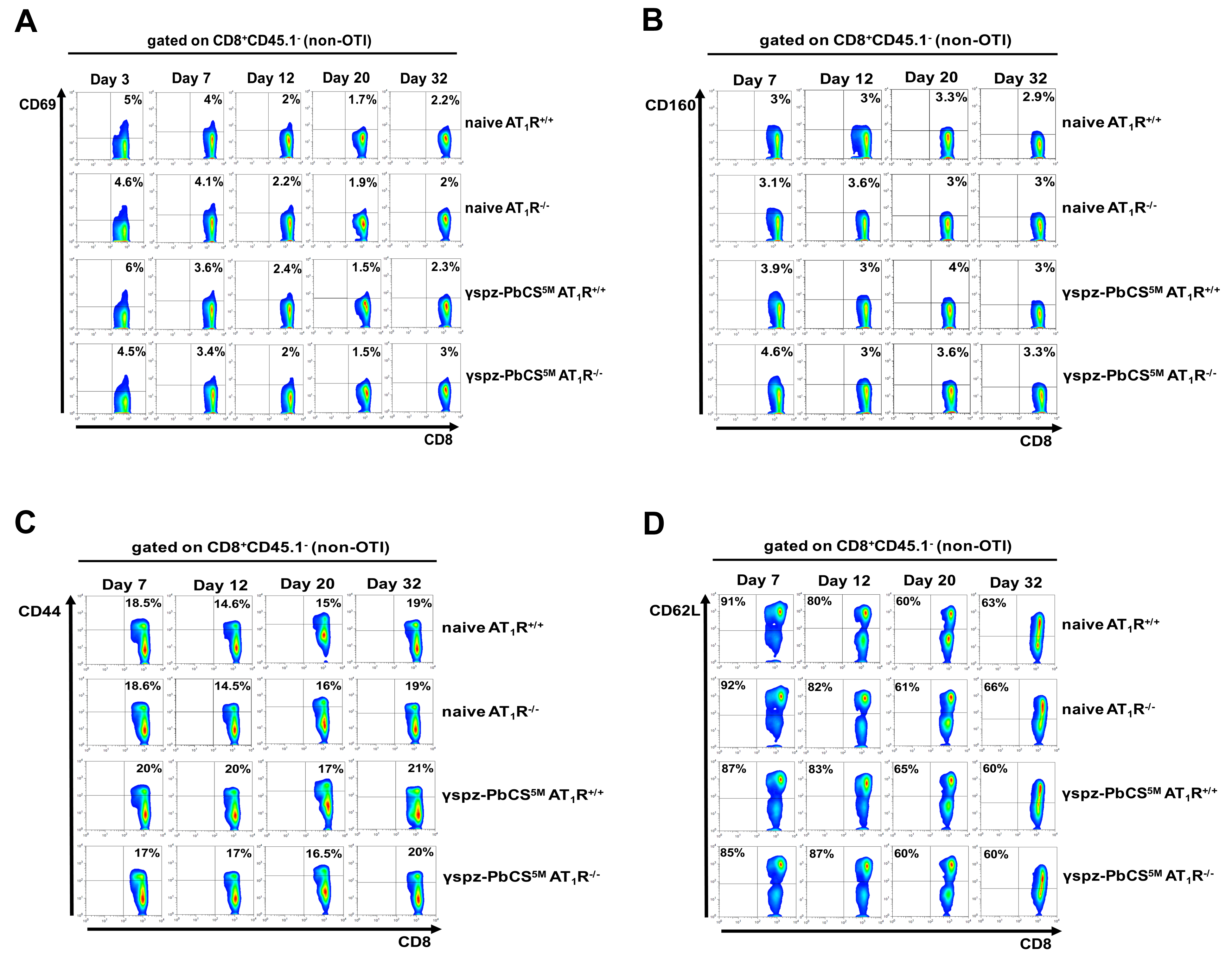


**Supplementary Figure S4. Activation of CD8^+^ T cells during γ-spz immunization occurs in an antigen specific-manner.**

For a better evaluation of effects of immunization (antigenic stimulation) on the antigen-specific CD8^+^ T cells, percentages of non-OT-I cells, i.e., the endogenous CD8^+^ T cells population (gated on CD8^+^CD45.1^-^ cells) in the recipient mice expressing the activation markers CD69, CD160, CD44 and CD62L were analyzed at days 0, 3, 7, 12, 20, and 32 post immunization.

(A, B, C, D) Representative dot plots of endogenous CD69^+^ (A), CD160^+^ (B), CD44^+^ (C) and CD62L^+^CD8^+^ T cells (D) in the spleen of AT_1_R^+/+^ or AT_1_R^-/-^ OT-I recipient mice (naïve or immunized with γ-spz of *P. berghei* CS^5M^) on days 3, 7, 12, 20 and 32 after immunization. Data are means of 4 mice per group and are pooled from 3 independent experiments with similar results for each indicated day.


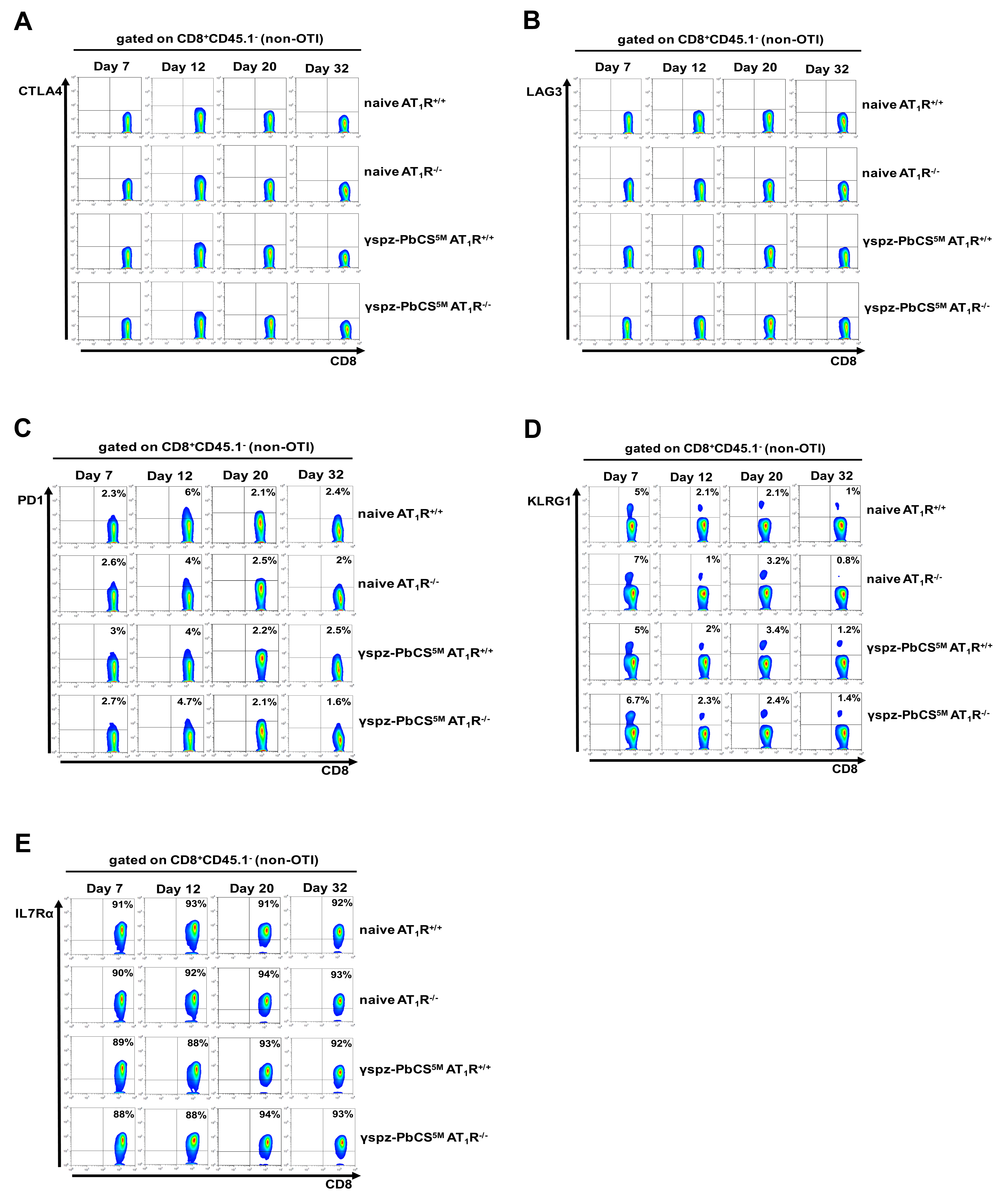


**Supplementary Figure S5. Changes in the expression of markers of exhaustion and memory in CD8^+^ T cells during γ-spz immunization occurs in an antigen specific-manner.**

For a better evaluation of effects of immunization (antigenic stimulation) on the antigen-specific CD8^+^ T cells, percentages of non-OT-I cells, i.e., the endogenous CD8^+^ T cells population (gated on CD8^+^CD45.1^-^ cells) in the recipient mice expressing the markers of exhaustion PD-1, LAG3 and CTLA4, and the memory markers KLRG1 and IL-7Rα were analyzed at days 0, 3, 7, 12, 20, and 32 post immunization.

(A, B, C) Representative dot plots of endogenous CTLA4^+^ (A), LAG3^+^ (B), PD1^+^ (C) in the spleen of AT_1_R^+/+^ or AT_1_R^-/-^ OT-I recipient mice (naïve or immunized with γ-spz of *P. berghei* CS^5M^) on days 3, 7, 12, 20 and 32 after immunization.

(D, E) Representative dot plots of endogenous KLRG1^+^ (D) and IL-7Rα (E) in the spleen of AT_1_R^+/+^ or AT_1_R^-/-^ OT-I recipient mice (naïve or immunized with γ-spz of *P. berghei* CS^5M^) on days 3, 7, 12, 20 and 32 after immunization.

Data are means of 4 mice per group and are pooled from 3 independent experiments with similar results for each indicated day.


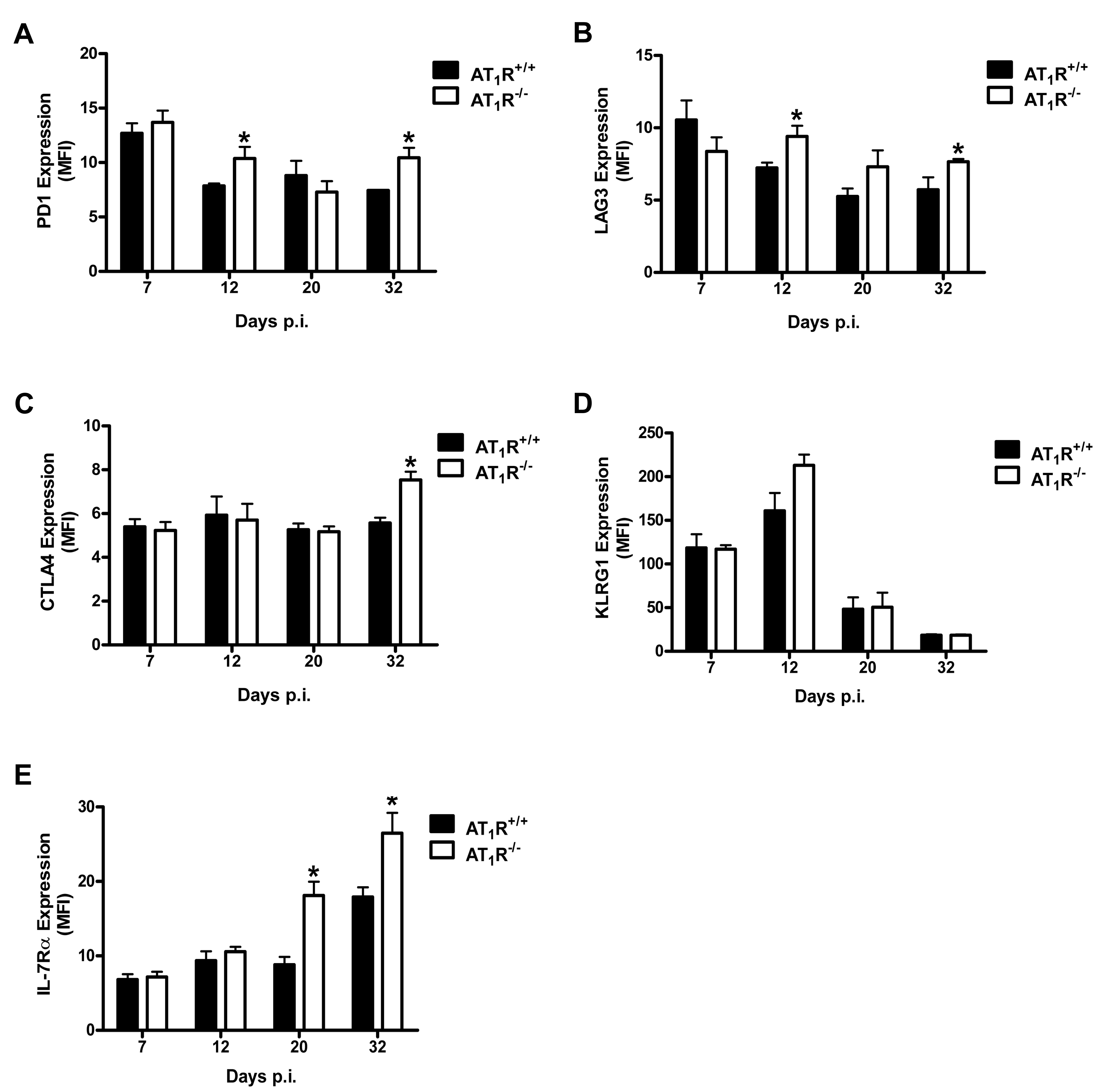


**Supplementary Figure S6. Higher expression of exhaustion markers and IL-7Rα in AT_1_R-deficient antigen-specific CD8^+^ T cells during the memory development phase.**

1 x 10^4^ naive AT_1_R^+/+^ or AT_1_R^−/−^ CD45.1^+^ OT-I cells were adoptively transferred to WT C57BL/6 (CD45.2^+^) recipients 1 day before intravenous inoculation with 1 × 10^5^ γ-irradiated *P. berghei CS^5M^* sporozoites.
(A) PD-1 (day 7, p = 0.5010; day 12, *p = 0.0415; day 20, p = 0.3849; day 32, *p = 0.04).
(B) LAG-3 (day 7, p = 0.2139; day 12, *p = 0.021; day 20, p = 0.2149; day 32, *p = 0.048).
(C) CTLA-4 (day 7, p = 0.7714; day 12, p = 0.8537; day 20, p = 0.8122; day 32, *p = 0.011) expression was evaluated, based on MFI analysis, gated in AT_1_R^+/+^ and AT_1_R^−/−^ OT-I cells (CD8^+^CD45.1^+^) recovered from the spleen of immunized recipient mice (CD45.2^+^) at indicated time points post immunization. Data are means ± SEM of 4 mice per group and are pooled from 2 independent experiments with similar results for each indicated day.

(D) KLRG-1 (day 7, p = 0.9348; day 12, p = 0.1465; day 20, p = 0.9280; day 32, p = 0.9848).
(E) IL-7Rα (day 7, p = 0.7406; day 12, p = 0.1072; day 20, *p = 0.0048; day 32, *p = 0.0136) expression was evaluated, based on MFI analysis, gated in AT_1_R^+/+^ and AT_1_R^−/−^ OT-I cells (CD8^+^CD45.1^+^) recovered from the spleen of immunized recipient mice (CD45.2^+^) at indicated time points post immunization. Data are means ± SEM of 4 mice per group and are representative of 2 independent experiments with similar results for each indicated time point.

Abbreviations: CTLA-4, cytotoxic T-lymphocyte-associated protein 4; LAG-3, lymphocyte-activation gene 3; PD-1, programmed cell death 1; IL7-Rα, interleukin-7 receptor α-chain; KLRG-1, killer cell lectin like receptor G1; MFI, median of fluorescence intensity.


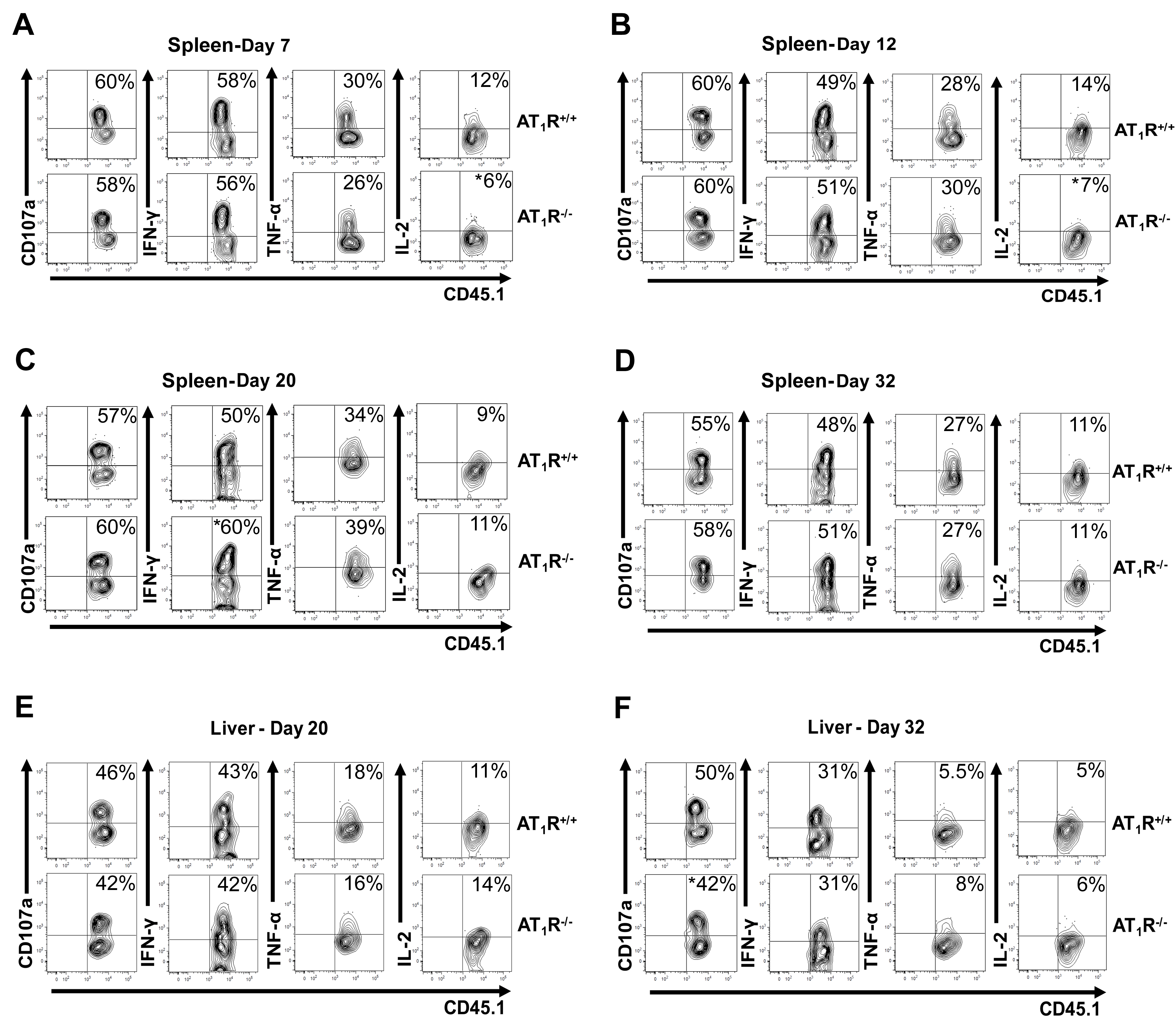


**Supplementary Figure S7. Characteristic staining of the populations of cells with cytotoxic capacity, measured by surface mobilization of CD107a, as well as producing cytokines (IFN-γ, TNF-α, and IL-2) determined after ex vivo re-stimulation with SIINFEKL-pulsed target cells at the different days post immunization.**

1 x 10^4^ naive AT_1_R^+/+^ or AT_1_R^−/−^ CD45.1^+^ OT-I cells were adoptively transferred to WT C57BL/6 (CD45.2^+^) recipients 1 day before intravenous inoculation with 1 × 10^5^ γ-irradiated *P. berghei CS^5M^* sporozoites. Dot plots are gated on CD8^+^CD45.1^+^ cells as indicated in the gating strategy in the Materials and Methods section. Numbers on dot plots indicate percentage of cells in the positive quadrant.

(A, B, C, D) 7 (A), 12 (B), 20 (C) and 32 days post immunization, spleen suspensions were stimulated ex vivo with SIINFEKL peptide-coated target cells. CD8^+^ T-cell functionality was determined by the combinatorial analysis of the CD8^+^CD45.1^+^ T cells producing CD107a (in cell surface), IFN-γ, TNF-α and IL-2. Data are means of 4 mice per group and are pooled from 3 independent experiments with similar results for each indicated day (*p < 0.05 in relation to AT_1_R^+/+^ OT-I cells).

(E, F) 20 (E) and 32 (F) days post immunization, liver suspensions were stimulated ex vivo with SIINFEKL peptide-coated target cells. CD8^+^ T-cell functionality was determined by the combinatorial analysis of the CD8^+^CD45.1^+^ T cells producing CD107a (in cell surface), IFN-γ, TNF-α and IL-2. Data are means of 4 mice per group and are pooled from 2 independent experiments with similar results for each indicated day (*p < 0.05 in relation to AT_1_R^+/+^ OT-I cells).
